# Claudin-5 binder enhances focused ultrasound-mediated opening in an *in vitro* blood-brain barrier model

**DOI:** 10.7150/thno.65539

**Published:** 2022-01-31

**Authors:** Liyu Chen, Ratneswary Sutharsan, Jonathan LF Lee, Esteban Cruz, Blaise Asnicar, Tishila Palliyaguru, Joanna M Wasielewska, Arnaud Gaudin, Jae Song, Gerhard Leinenga, Jürgen Götz

**Affiliations:** 1 Clem Jones Centre for Ageing Dementia Research, Queensland Brain Institute, The University of Queensland, Brisbane (St Lucia Campus), QLD 4072, Australia.; 2 Cell and Molecular Biology Department, Mental Health Program, QIMR Berghofer Medical Research Institute, Brisbane, QLD 4006, Australia.; 3 Advanced Microscopy Facility, Queensland Brain Institute, The University of Queensland, Brisbane (St Lucia Campus), QLD 4072, Australia.

**Keywords:** Blood-brain barrier (BBB), claudin-5, focused ultrasound, transendothelial electrical resistance (TEER)

## Abstract

**Rationale:** The blood-brain barrier (BBB) while functioning as a gatekeeper of the brain, impedes cerebral drug delivery. An emerging technology to overcome this limitation is focused ultrasound (FUS). When FUS interacts with intravenously injected microbubbles (FUS^+MB^), the BBB opens, transiently allowing the access of therapeutic agents into the brain. However, the ultrasound parameters need to be tightly tuned: when the acoustic pressure is too low there is no opening, and when it is too high, tissue damage can occur. We therefore asked whether barrier permeability can be increased by combining FUS^+MB^ with a second modality such that in a clinical setting lower acoustic pressures could be used.

**Methods:** Given that FUS^+MB^ achieves BBB opening in part by disruption of tight junction (TJ) proteins such as claudin-5 of brain endothelial cells, we generated a stable MDCK (Madin-Darby Canine Kidney) II cell line (eGFP-hCldn5-MDCK II) that expresses fluorescently tagged human claudin-5. Two claudin-5 binders, the peptide mC5C2 and cCPEm (truncated form of an enterotoxin), reported previously to weaken the barrier, were synthesized and assessed for their abilities to enhance the permeability of cellular monolayers. We then performed a comparative analysis of single and combination treatments, measuring transendothelial electrical resistance (TEER) and cargo leakage, combined with confocal image analysis.

**Results:** We successfully generated a novel cell line that formed functional monolayers as validated by an increased TEER reading and a low (< 0.2%) permeability to sodium fluorescein (376 Da). We found that the binders exerted a time- and concentration-dependent effect on barrier opening when incubated over an extended period, whereas FUS^+MB^ caused a rapid opening followed by recovery after 12 hours within the tested pressure range. Importantly, preincubation with cCPEm prior to FUS^+MB^ treatment resulted in greater barrier opening compared to either FUS^+MB^ or cCPEm alone as measured by reduced TEER values and an increased permeability to fluorescently labelled 40 kDa dextran (FD40).

**Conclusion:** The data suggest that pre incubation with clinically suitable binders to TJ proteins may be a general strategy to facilitate safer and more effective ultrasound-mediated BBB opening in cellular and animal systems and potentially also for the treatment of human diseases of the brain.

## Introduction

One of the biggest challenges in developing therapeutics for central nervous system (CNS) disorders is to achieve sufficient blood-brain barrier (BBB) penetration 1. It has been estimated that the BBB presents an obstacle for more than 98% of small-molecule drugs and nearly all large-molecule therapeutics for CNS disorders 2. However, the use of low-intensity focused ultrasound (FUS) in combination with intravenously injected microbubbles (FUS^+MB^) is currently the only targeted, non-invasive technique that can transiently disrupt the BBB to allow access of therapeutics to the brain 3. The FUS^+MB^ technology has been shown in multiple studies to deliver a wide range of different molecules in both small and large animal models, and several clinical trials targeting brain tumours or neurodegeneration have been conducted or launched 4-13. This has been driven by the notion that to effectively treat diseases such as brain cancer or Alzheimer's disease (AD), the drug concentrations reaching the brain need to be significantly increased. The applied ultrasound parameters and the ensuing effect on the circulating microbubbles determines the extent and duration of BBB opening and hence, drug uptake 14, 15. When the pressure is low, the microbubbles oscillate in a radial direction (i.e., they expand and contract), a process defined as stable cavitation 16. When the pressure is increased, this not only leads to enhanced BBB opening, but the microbubbles also undergo inertial cavitation which causes them to collapse, inducing micro-jetting and shock emission. Because of the strong mechanical effects of inertial cavitation, this results in tissue damage, in particular microhemorrhages and oedemas 17. While neither the threshold pressure differentiating the two regimes (stable versus inertial cavitation) is clearly defined nor how stable cavitation is accurately related to BBB opening, this suggests that lowering the threshold pressure required for BBB opening would reduce or even eliminate inertial cavitation-related damage 18, 19. In other words, such an approach would enhance the extent of BBB opening without requiring high pressure sonications. We therefore investigated, using a novel *in vitro* BBB model, whether permeability can be increased by combining FUS with molecules that bind to and alter the properties of tight junctions (TJs) that make up the BBB.

**Figure 1:**
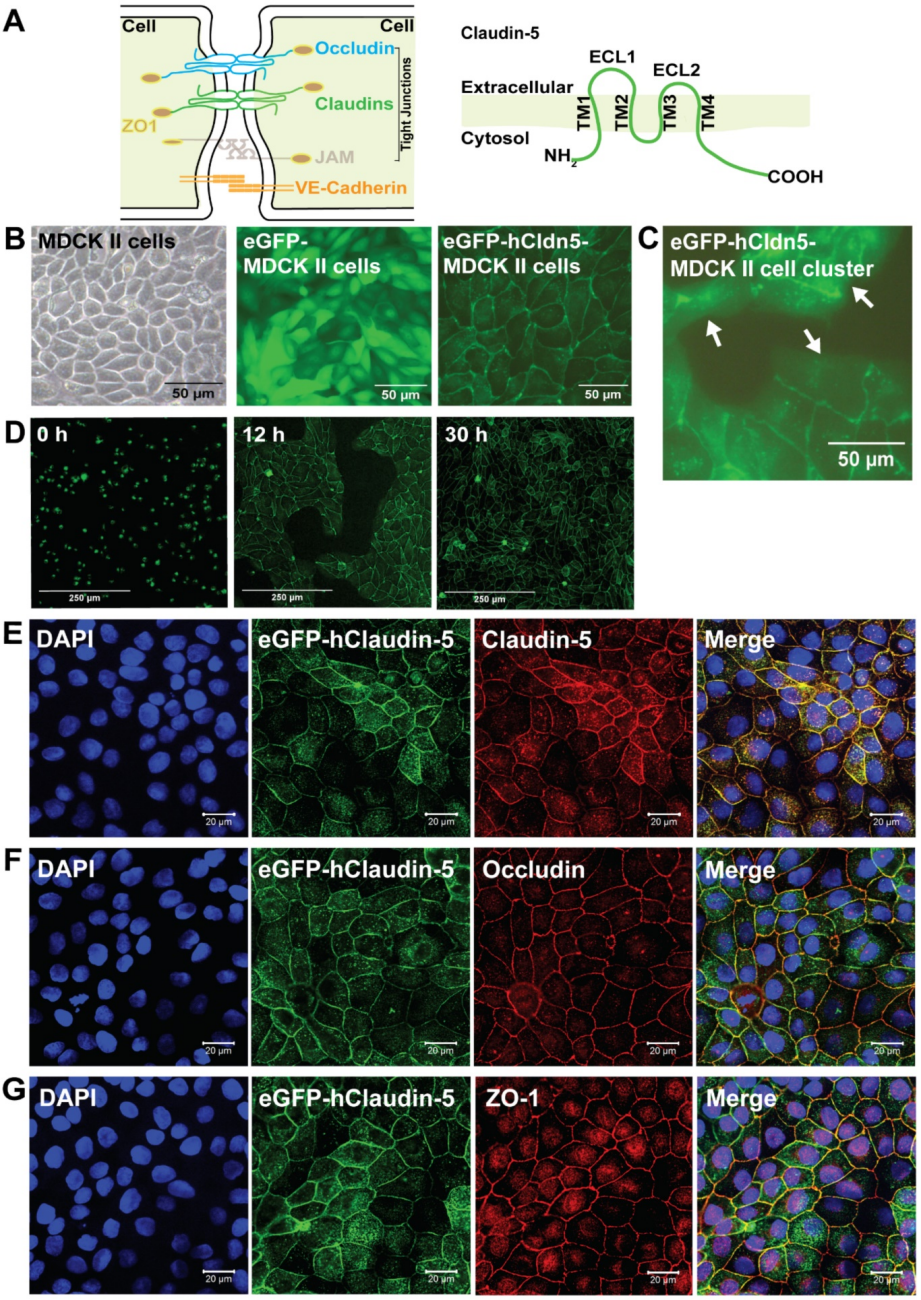
** eGFP-hCldn5-MDCK II cells exhibit a tight monolayer and human claudin-5 is localized to cell/cell contacts. (A)** Scheme of tight junctions formed by proteins such as claudin-5, occludin, and ZO-1. Domain structure of claudin-5. **(B)** Representative phase contrast image of confluent parental MDCK II cells and epifluorescence images of confluent MDCK II cells expressing eGFP only (eGFP-MDCK II) or eGFP-tagged human claudin-5 (eGFP-hCldn5-MDCK II). **(C)** Epifluorescence images of isolated clusters of eGFP-hCldn5-MDCK II cells. White arrows indicate the absence of localization of eGFP fluorescence in areas without cell/cell contacts. **(D)** Time-lapse fluorescence images of eGFP-hCldn5-MDCK II cells from the time of seeding at a density of 200,000 cells/cm^2^ (0 h) to complete formation of a confluent monolayer (30 h). **(E)** Expression of claudin-5, **(F)** occludin and **(G)** ZO-1, localized to cell/cell borders. Nuclei were stained with DAPI. Scale bars: 50 µm (B-C), 250 µm (D) and 20 µm (E-G).

At the cellular level, the BBB is represented by the neurovascular unit (NVU) that includes endothelial cells, pericytes and astrocytes, as well as an ensheathing basement membrane. The NVU generates a dynamic interface between the circulation and brain parenchyma, providing anatomical and physiological protection for homeostasis of the CNS 1, 20, 21. Transport across the brain endothelium is tightly controlled via (i) a paracellular barrier represented by interendothelial TJs; (ii) a transcytoplasmic barrier, owing to the unique properties of the brain capillary endothelial cells (BCECs) with a low level of endo- and transcytosis; (iii) an enzymatic barrier; and (iv) active multidrug efflux transporters. FUS^+MB^ achieves transient BBB opening both by facilitating transcytoplasmic transport and by severing the homophilic interactions between TJ proteins 22, 23. The TJs of BCECs are formed by a group of proteins which includes occludin, ZO-1 and the claudin family, of which claudin-5 is the most highly expressed 24 **(Figure 1A)**. Claudin-5 has four transmembrane domains, a short cytoplasmic amino-terminus, two extracellular loops (ECL1 and ECL2), an intracellular loop and a longer cytoplasmic carboxy-terminus **(Figure 1A)**. The ECLs contribute to homo- and heterophilic interactions, thereby tightening the paracellular space 25, 26.

Several *in vitro* models which recapitulate critical physiological parameters and molecular aspects of the NVU are available for studying the BBB. Analysis tools include immunolabelling of TJ proteins, and when cultured in Transwell inserts, allows measurement of the transendothelial electrical resistance (TEER) and leakage of cargoes 27. TEER values are strong indicators of the integrity of cellular barriers before they are evaluated for transport of drugs or chemicals 28. The TEER values measured *in vivo* have been reported to be as high as 5,900 Ω·cm^2^ 29. In a co-culture system with human induced pluripotent stem cell (hiPSC)-derived endothelial cells (iBECs) and neuronal progenitor cells treated with retinoic acid, TEER values of up to 5,000 Ω·cm^2^ have been achieved 30. However, having an ongoing supply of iBECs is both costly and challenging 31. On the other hand, cell lines such as immortalized human cerebral microvascular endothelial cells (hCMEC/D.3), co-cultured with astrocytes, yield a relatively low TEER value of 140 Ω·cm^2^ 32. There are not many non-human cerebral endothelial cell lines available. The murine cerebral endothelial cell line (bEnd.3) exhibits an even lower TEER of < 50 Ω·cm^2^, which is far from ideal to display sufficient tightness as an *in vitro* BBB permeability model 33, 34. Here, we generated a Madin-Darby Canine Kidney (MDCK II) cell line that stably expresses fluorescently tagged human claudin-5 and has around three-fold higher TEER values than the parental cell-line, to assess BBB weakening and cargo leakage in response to different interventions.

Given the central role of claudin-5 in the NVU, several peptide-based approaches have been previously developed to target claudin-5 and thereby weaken the BBB. Dithmer and colleagues designed peptides based on the ECL1 domain 35. Of these, peptide mC5C2 displayed a nanomolar affinity for claudin-5 and achieved a size-selective (up to 40 kDa dextran) and reversible (12-48 h) increase in paracellular transport of cargoes across brain endothelial and claudin-5-transfected epithelial cell monolayers. Furthermore, the peptide safely opened the murine BBB *in vivo*, as demonstrated by leakage of a contrast agent using magnetic resonance imaging.

Different from mC5C2, the non-toxic carboxy-terminal domain of the clostridium perfringens enterotoxin, cCPE, binds to ECL2 of the subtype-specific claudins, claudin-3 and claudin-4, with high affinity, increasing paracellular permeability and enhancing drug absorption 36, 37. Because cCPE does not bind to claudin-5, Protze and colleagues performed site-directed mutagenesis and generated a novel cCPE mutant (cCPE_Y306W/S313H_, cCPEm) previously shown to achieve a nanomolar affinity for claudin-5 as well as transient BBB opening by selectively binding to claudin-5 38. cCPEm decreased TEER in a concentration-dependent and reversible manner in three *in vitro* BBB models derived from three species 39.

Here, we investigated the effects of FUS^+MB^ and the two claudin-5 binders, mC5C2 and cCPEm, both alone and in combination, on BBB properties in an MDCK II cell culture model we had generated that expresses fluorescently tagged human claudin-5. Our results revealed that pre incubation with cCPEm improves the degree of opening for a range of acoustic pressures below 0.3 MPa (required for barrier opening) as determined by TEER and leakage of fluorescently labelled cargoes. We also performed spinning disk confocal microscopy and used the 3D segmentation software Imaris to quantify the relocation of the eGFP-hCldn5 signal from the membrane to the cytosol across experimental conditions. We anticipate in experimental animal models as well as in a clinical setting that intravenous injection of carefully selected binders of either TJ or adherens junction proteins prior to the application of FUS^+MB^ may reduce the pressures required to achieve safe BBB opening and hence, improve therapeutic outcomes.

## Materials and methods

### Claudin-5 peptidomimetics and generation of the GST-cCPEm fusion protein

The claudin-5 peptidomimetic mC5C2 corresponding to residues 53-81 of ECL1 of murine claudin-5 was synthesized by GeneScript (Piscataway, New Jersey, United States), using standard solid-phase peptide synthesis and fluorenylmethyloxycarbonyl (Fmoc) chemistry on a preloaded Fmoc-amino acid resin 35. The peptide was carboxy-terminally amidated and had the expected mass of 3095 Da and a purity >95%.

The glutathione S-transferase (GST) cCPEm fusion protein (**Figure S1**) was generated by subcloning the carboxy-terminal domain of CPE (aa 194-319) with two mutations Y306W and S313H previously shown to achieve a nanomolar affinity for claudin-5 38) via the *EcoRI* and *SalI* restriction sites of the pGEX-4T1 (GE Healthcare, Cincinnati, Ohio, United States, cat. #28-945-9545-49) plasmid, in-frame with an amino-terminal GST tag to facilitate protein purification, thereby generating plasmid pGEX-4T1-cCPEm.

Next, BL21(DE3) *E. coli* (New England Biolabs, Ipswich, Massachusetts, United States, cat. #C25271) were transformed with pGEX-4T1-cCPEm and grown to OD_600_ ~ 0.6, with expression being induced by adding isopropy1-β-D-thiogalactopyranoside (IPTG, Meridian Bioscience, Cincinnati, Ohio, United States, cat. #BIO-37036) to a final concentration of 1 mM. After 6 h at 30 ºC with constant vigorous shaking, the bacteria were harvested by centrifugation (6,000 g, 20 min, 4 ºC) and stored at -80 ºC until purification. The bacterial pellet was resuspended in lysis buffer (25 mM tris-HCl, pH 8; 150 mM NaCl, 5 µM 1,4-dithiothreitol) (DTT) containing protease inhibitor (Complete Mini, EDTA-free; Roche Applied Science, Mannheim, Germany), 10 U/ml benzonase nuclease (Sigma-Aldrich, St. Louis, Missouri, United States, cat. #E1014) and 100 µg/ml lysozyme (Amresco, Solon, Ohio, United States, cat. #0663-10G), and incubated on ice for 20 min. Cells were then lysed on ice by intermittent sonication using the Sonics Vibra-Cell VCX130 sonicator (Newtown, Connecticut, United States) with an output power of 130 Watts and a 20 kHz pulse frequency at a 60% amplitude for 3 min. The cell lysates were centrifuged at 20,000 g for 30 min at 4 ºC and the supernatant was filtered through a 0.22 µm syringe filter (Millipore, Billerica, Massachusetts, United States).

Purification of the GST-tagged recombinant protein was performed as described previously 40, 41. Briefly, the supernatant of the bacterial lysate was purified by affinity chromatography using an automated Profinia Protein Purification system (Bio-Rad, Hercules, California, United States), followed by size-exclusion chromatography using a Superdex 200 Increase 10/300 GL column (GE Healthcare, cat. #28990944) with an ÄKTApurifier chromatography system (GE Healthcare) in 1× phosphate-buffered saline (PBS) with 1 mM DTT. A_280_ peak fractions were collected and assayed for GST expression by Coomassie staining and immunoblotting with an anti-GST antibody. GST-cCPEm containing fractions were pooled. The protein concentration was determined using a NanoDrop 2000 spectrophotometer (Thermo-Fisher, Wilmington, United States) using a molar extinction coefficient of 73,185 M^-1^cm^-1^, as calculated by the online Expasy ProtParam portal 42. Aliquots of GST-cCPEm were stored at 80 ºC until use.

### Molecular cloning, lentiviral particle production and generation of eGFP-hCldn5-MDCK II cells

To generate an MDCK II cell line that expresses eGFP-tagged human claudin-5 (hCldn5), a lentiviral vector, pLV-eGFP-hCldn5, was generated. First, a gBlock^TM^ double-stranded DNA fragment was generated for hCldn5, which included an amino-terminal glycine/serine-rich linker sequence. The gBlock was then amplified by PCR to introduce the *KpnI and MluI* restriction sites. Next, eGFP was amplified from plasmid pLV-eGFP (Addgene, Cambridge, Massachusetts, United States, cat. #36083), and the flanking *NheI* and *KpnI* restriction sites were introduced. The amplified products were digested and ligated with T4 DNA ligase (New England Biolabs cat. #B0202S) into pLX_311-KRAB-dCas9 (Addgene, cat. #96918) using the *NheI* and *MluI* restriction enzymes to generate pLX-eGFP-hCldn5, followed by sequencing for verification purposes. As a negative control, we constructed an empty eGFP lentiviral vector (pLX-eGFP).

The experimental procedure to generate active lentiviral particles has been published previously 43. Briefly, adherent Lenti-X 293T cells (Takara Bio, Tokyo, Japan, cat. #632180) were cultured in Dulbecco's Modified Eagle Medium (DMEM) with pyruvate containing 10% fetal bovine serum (FBS). The third generation lentiviral packaging system plasmids pMDLg/pRRE (Addgene, cat. #12251), pRSV-Rev (Addgene, cat. #12253) and envelope expressing plasmid pMD2.g (Addgene, cat. #12259) were used to transfect Lenti-X 293T cells by CaPO_4_ precipitation 44, 45. The lentivirus-containing medium was collected after 48 and 72 h, centrifuged at 3,000 g for 5 min and filtered through a 250 ml 0.45 µm bottle-top vacuum filter. For the final purification and concentration, the lentivirus-containing medium was suspended above a 10% sucrose cushion (100 mM NaCl, 0.5 mM EDTA, 10% sucrose, 50 mM Tris-HCl to pH 7.4) and centrifuged at 10,000 g for 4 h at 4 °C. The supernatant was discarded, the lentiviral pellets were resuspended in 100 µl of 1× HBSS (Hank's balanced salt solution, Thermo-Fisher, cat. #14175095), and 20 µl aliquots were snap-frozen in liquid nitrogen and then stored at -80 °C until use. As a negative control, lentiviral particles packaged with pLX-eGFP were generated.

To generate a stable eGFP-hCldn5-MDCK II cell line, MDCK II cells were transduced with active lentiviral particles for 72 h. Infected cells were then exposed to growth medium containing 8 µg/ml blasticidin (Sigma-Aldrich, cat. #15205) for up to 14 days (**Figure S2**).

### Cell culture and additional cell-lines

MDCK II cells were purchased from the European Collection of Authenticated Cell Culture (ECACC, cat. #00062107) and used to generate a cell line that stably expresses human claudin-5 amino-terminally tagged with eGFP. All MDCK II cell lines in this study were cultured in Minimum Essential Medium Eagle (MEM) (Sigma-Aldrich, cat. #M4655) supplemented with 5% FBS. Lenti-X 293T cells were used for lentiviral particles production and cultured in DMEM or DMEM supplemented with 1 mM pyruvate (Thermo-Fisher, cat. #11995073) and 10% FBS. All culture media contained 100 U/ml penicillin and 100 U/ml streptomycin (Thermo-Fisher, cat. #15070063). Dulbecco's phosphate-buffered saline (DPBS, Thermo-Fisher, cat. #14190144) and Hanks' Balanced Salt Solution with Ca^2+^ and Mg^2+^ (HBSS 1X, Gibco, cat. #14025-092) were used for washing steps. All cell lines were maintained in 5% CO_2_ at 37 ºC.

iBECs were derived from hiPSCs. hiPSCs, a generous gift provided by Dr. Anthony White (QIMR Berghofer Medical Research Institute, Australia) were maintained on human recombinant vitronectin in StemFlex medium (Life Technologies, Carlsbad, California, United States, cat. # A3349401). Differentiation was performed as previously described 31, 46. Briefly, cells were plated on Matrigel (*In vitro* Technologies, cat. # FAL354277) coated plates in StemFlex medium supplemented with 10 µM ROCK inhibitor (StemCell Technologies, Vancouver, British Columbia, Canada, cat. #. 72304). Three days after plating, the culture medium was changed for unconditioned medium consisting of DMEM/F12+GlutaMAX, 20% Knockout Serum Replacement (Life Technologies, cat. #.10828028), 1× non-essential amino acids (Sigma-Aldrich, cat. #M7145) and 0.1 mM β-mercaptoethanol (Sigma-Aldrich, cat. #M3148-25ml) to induce spontaneous differentiation. After 6-8 days in unconditioned medium, the culture medium was changed to endothelial cell medium (ECM) supplemented with 2% B27 (Life Technologies, cat. #17504-044), 20 ng/ml basic fibroblast growth factor (bFGF) (Peprotech, cat. #100-18B-250) and 10 µM retinoic acid (Sigma-Aldrich, cat. #R2625). Cells were maintained in supplemented ECM for 2-3 days, after which they were transferred onto collagen IV (Sigma-Aldrich, cat. #C5533) and fibronectin (Life Technologies, cat. #33016015) coated Transwell inserts (Corning Inc., New York, United States, cat. #CLS3470). After a further 2 days, the cells were maintained in ECM+B27 medium without bFGF and retinoic acid for one additional day. TEER reading was performed on these iBECs 48 h after subculturing, with cells displaying 100% confluency.

### Validation of GST-cCPEm binding to claudin-5 in isolated cerebral microvessels

To validate GST-cCPEm binding to endogenous claudin-5, cerebral microvessels were isolated from C57Bl6/J mice, with modification of a previously published protocol 47. Briefly, animals were euthanized by intraperitoneal injection of 350 mg/kg pentobarbitone (Lethabarb, Virbac), after which their brains were dissected and submerged in MCDB131 medium (Thermo-Fisher, cat. #10372019). Meninges and meningeal vessels were removed by gently rolling the brains on damp blotting paper (Thermo-Fisher, cat. #14190144). The grey matter was dissected out using a razor blade and curved forceps, followed by Dounce homogenization in MCDB131 medium using a tissue grinder. The suspension was centrifuged at 2,000 g for 5 min at 4 ºC (Beckman Coulter, Krefeld, Germany, Avanti J-26 XPI) to remove myelin debris. The pellet was then resuspended in Dulbecco's phosphate buffered saline (DPBS) containing 25% w/v 70 kDa dextran, and then centrifuged at 10,000 g for 15 min at 4 ºC to sediment the red microvessel pellet. The step was repeated for further white matter removal. The pellets were resuspended in MCDB131, transferred to 40 µm cell strainers, and washed with DPBS. The filters were then inverted and microvessels were retrieved with MCDB131 solution containing 0.5% bovine serum albumin (BSA). Cellular composition and purity were validated for expression of glial fibrillary acidic protein (GFAP) and platelet derived growth factor receptor β (PDGFRβ) to confirm the presence of astrocytes and pericytes, respectively, and of class III β-tubulin (TUJ1) to confirm the absence of neurons.

To determine GST-mCPE binding, the purified microvessels were plated onto Superfrost Plus microscope slides (Menzel-Gläser, Braunschweig, Germany, cat. #SF41296SP) and left to air-dry at room temperature for 30 min. They were then incubated with 100 µg/ml GST-cCPEm diluted in MCDB131 + 0.5% BSA in a 37 ºC incubator for 30 min, followed by three washes with DPBS containing 0.1% NP-40. They were then fixed with 4% paraformaldehyde (PFA)-DPBS for 15 min, permeabilised with DPBS + 0.1% NP-40 for 15 min and blocked with 5% BSA-DPBS at room temperature for 1 h. The microvessels were subsequently incubated with an anti-mouse GST primary antibody at 4 ºC overnight, washed three times with DPBS + 0.1% NP-40, stained with a goat-anti-mouse Alexa Fluor 594 secondary antibody for 1 h at room temperature, and counterstained with 4′,6-diamidino-2-phenylindole (DAPI). Slides were mounted with Vectashield antifade mounting medium and images captured using a Plan Apochromat 63×/1.4 NA oil-immersion objective on a Zeiss LSM 710 confocal microscope built around a Zeiss Axio Observer Z1.

### Dextran and sodium fluorescein cargoes

Fluorescein isothiocyanate-dextrans (FDs) and sodium fluorescein (NaFl) were used for permeability studies. FD40 has a molecular weight of 40 kDa (Thermo-Fisher, cat. #D1845) with a hydrodynamic radius (R_H_) of ~6 nm. The dextrans were diluted to a stock concentration of 10 mg/ml in PBS. NaFl (Sigma-Aldrich, cat. #F6377) with a molecular weight of 376 Da was diluted to a stock concentration of 30 mM in PBS.

### Antibodies

Primary antibodies for immunocytochemistry were for claudin-5 (Thermo-Fisher, cat. #34-1600, 1:250), occludin (Thermo-Fisher, cat. #40-4700, 1:250), ZO-1 (Thermo-Fisher, cat. #61-7300, 1:500), CD31 (Abcam, Cambridge, United Kingdom, cat. #ab28364, 1:200), GFAP (Covance, Princeton, New Jersey, United States, cat. #MAB360, 1:200), PDGFRβ (Cell Signalling, Beverly, Massachusetts, United States, cat. #28E1, 1:200), TUJI (Covance, cat. MMS-435P, 1:200) and the GST tag (Proteintech, Chicago, Illinois, United States, cat. #66001-2-1g, 1:500). Secondary antibodies were goat-anti-mouse Alexa Fluor 488 (Thermo-Fisher, cat. #A-11029, 1:500), goat-anti-mouse Alexa Fluor 594 (Thermo-Fisher, cat. #A-11005, 1:500), goat-anti-rabbit Alexa Fluor 488 (Thermo-Fisher, cat. #A-1108, 1:500) and goat-anti-rabbit Alexa Fluor 647 (Thermo-Fisher, cat. #A-21245, 1:500). Primary antibodies for western blotting were for claudin-5 (Thermo-Fisher, cat. #34-1600, 1:1,000), occludin (Thermo-Fisher, cat. #40-4700, 1:1,000), ZO-1 (Thermo-Fisher, cat. #61-7300, 1:1,000), GFP (Aves Labs, Tigard, Oregon, United States, cat. #GFP-1020, 15,000) and the GST tag (Proteintech, cat. #66001-2-1g, 1:2000). Secondary antibodies were IRDye^®^ 800CW goat-anti-mouse (Li-Cor, Cambridge, United Kingdom, cat. #926-32210, 1:10,000), IRDye^®^ 680LT goat anti-rabbit IgG (H + L) (Li-Cor, cat. #926-6802, 1:10,000) and horseradish peroxidase (HRP)-conjugated goat anti-chicken IgG (GE Healthcare, cat. #AS09603, 1:10,000).

### Immunocytochemistry

The cells were grown on coverslips and washed twice with cold HBSS, followed by fixation in 4% PFA for claudin-5 and ZO-1 staining and ice-cold 98% ethanol for occludin staining, respectively. Cells were then washed with 0.1 M glycine in PBS or ice-cold PBS, respectively, followed by permeabilization in 0.1% Triton X-100 in PBS for 10 min at 4 ºC. Then, cells were pre-incubated in a blocking solution containing 5% goat serum and 1% BSA in PBS for 1 h. Samples were then incubated with primary antibodies overnight at 4 ºC on a rocker. Reactions were visualized with fluorescently labelled anti-rabbit antibodies. Images for Figure 1 were captured using a plan apochromat 63× oil objective (NA=1.4) on a Zeiss LSM 510 META confocal microscope, while all other images were acquired on a Yokogawa W1 spinning disk confocal microscope. eGFP-hCldn5, claudin-5, occludin and ZO-1 were acquired under consistent conditions (laser power, camera, optics and excitation time). Then, all acquired images were exported as tiff files to Huygens pro 20.10 (SVI) for deconvolution. These deconvolved images were then imported into Imaris 9.7.2 for segmentation. The ZO-1 signal was used to represent the plasma membrane as a surface. The eGFP-hCldn5 signal was detected as spots (for distance to membrane statistics) and surface (for intensity statistics).

### Western blotting

Confluent cells in T25 flasks were washed twice with PBS, and then lysed with 1× radioimmunoprecipitation assay (RIPA) buffer (Cell Signalling Technologies, Beverly, Massachusetts, United States, cat. #9806) and 200 mM phenylmethylsulfonyl fluoride (Sigma-Aldrich, cat. #P7626) with 1× protease inhibitor cocktail (Roche) for 20 min on ice. Lysates were then homogenized by sonication for 40 s (4× at 20% amplitude, 10 s each) and centrifuged at 10,000 × *g* for 20 min at 4 °C to remove cellular debris before the supernatant was collected for analysis. Total protein content was determined with the Pierce™ bicinchoninic acid protein (BCA) (Thermo-Fisher) assay kit, and samples were prepared by denaturing 15 µg of protein with 8 µl of 5× Laemmli buffer (60 mM Tris/Cl (pH 6.8), 2% SDS, 10% glycerol, 5% ß-mercaptoethanol and 0.01% bromophenol blue) at 95 °C for 5 min. Samples were then loaded onto 4-15% Criterion^™^ TGX^™^ Precast Midi Protein Gels (12 + 2 wells, 45 µl) (Bio-Rad) and subjected to SDS-PAGE at 200 V for ~45 min in 10× Tris/Glycine/SDS running buffer (Bio-Rad). Proteins were then transferred onto low-fluorescence 0.45 µm PVDF membranes with transfer buffer (10× Tris/glycine with 10% methanol) (Bio-Rad) for 10 min using the semi-dry Trans-Blot Turbo Transfer System (Bio-Rad). Non-specific binding was blocked with Odyssey^®^ Blocking Buffer (Li-Cor) or 5% skim milk in Tris-buffered saline (TBS) containing 0.1% Tween-20 for 1 h at room temperature followed by incubation with primary antibodies overnight at 4°C. Membranes were then washed 4 times (5 min each) before incubation with fluorescently labelled secondary antibodies or HRP-conjugated secondary antibodies for 1.5 h at room temperature. Following extensive washes, blots were visualized with an Odyssey Infrared Imager CLX (Li-Cor) and Image Studio™ software (Li-Cor).

### Microbubbles

Biologically inert microbubbles were prepared in-house as described previously 48, 49. Briefly, 1,2-distearoyl-*sn*-glycero-3-phosphocholine (DSPC) and 1,2-distearoyl-*sn*-glycero-3-phosphoethanolamine-N- [amino (polyethylene glycol)-2000] (DSPE-PEG2000) (Avanti Polar Lipids Inc., Alabaster, Alabama, United States) were mixed in a 9:1 molar ratio and dissolved in a small amount of chloroform (Sigma-Aldrich) in a glass beaker, followed by evaporation under vacuum (Mivac Quattro, Genevac Ltd, Ipswich, Suffolk, United Kingdom) at 22 °C for 20 min. The dried lipid film was then rehydrated in PBS with 10% glycerol to a concentration of 1 mg lipid/ml and heated to 55 °C in a sonicating water bath until fully dissolved. The clear solution was dispensed aseptically into 1.5 ml glass high-performance liquid chromatography (HPLC) vials and the air in each vial was replaced with octafluoropropane (Arcadophta). On the day of the experiment, the HPLC vial was brought to room temperature one hour ahead of the experiment. An equal volume of 0.9% NaCl solution was injected into the vial, followed by agitation in a dental amalgamator for 45 s to generate the MBs. The MBs were characterized for their size and concentration using a Multisizer 4e coulter counter (Beckman Coulter) as previously described 50.

### FUS application

Cells were cultured on Transwell culture inserts with a pore size of 0.4 μm which were placed into a 24-well plate. The plate was then placed on top of a Sonic Concepts H117 ultrasound transducer that was submerged in a water bath adjusted to 24 °C. A proper alignment of the focus was performed. 20 µl of microbubbles were added to the top chamber of each Transwell insert and exposed to FUS (286 kHz centre frequency, 50 cycles/burst, burst period 20 ms and a 120 s sonication time). A range of pressures (0.1, 0.2, 0.3 and 0.4 MPa) were tested and the input power was based on calibration of the system with a calibrated needle hydrophone (NPL, UK). For GST-cCPEm treatment, cells were incubated in a 37 °C incubator prior to FUS^+MB^ treatment.

### Transendothelial electrical resistance and paracellular permeability

TEER across cellular monolayers was measured with chopstick electrodes in 24-well Transwell inserts using the Millicell^®^ ERS-2 Voltohmmeter (Merck Millipore). The absolute TEER of eGFP-hCldn5-MDCK II cells before treatment was recorded as a baseline reading. Resistance of the blank filter was subtracted and then multiplied by the surface area of the membrane for calculation of the final TEER values. All experiments were carried out in triplicates, and data are expressed as mean ± SEM of two independent experiments. The permeability to fluorescently labelled tracer molecules was also used to estimate the paracellular opening in monolayers due to treatments. 10 µM NaFl or 0.5 mg/ml FD40 was added to the apical chambers and incubated at 37 ºC. After 2 h, 100 µl samples from the bottom chambers were collected for reading in a fluorescence plate reader (CLARIOstar-BMG Labtech) at 483 ± 14 nm excitation and 530 ± 30 nm emission wavelengths.

### Viability assays

To determine cell viability post-treatment, a cytotoxicity assay (CellTiter 96, Promega) was performed following the manufacturer's instructions. For all experimental conditions, premixed MTT (3-(4,5-dimethylthiazol-2-yl-2,5-diphenyltetrazoliumbromide; 0.25 mg/ml, Thermo-Fisher, cat. # M6494) solution was added to the wells, followed by incubation for 50 min. DMSO was then added to the culture wells, and the plate was incubated at room temperature on a rocker for 10 min. Absorbance was recorded at 570 nm using a plate reader (CLARIOstar-BMG Labtech). Values were measured as fold change compared to the untreated samples and plotted as mean ± SEM.

### Statistical analysis

Statistical analysis was performed on GraphPad Prism version 9.0.0 (GraphPad Software, San Diego, California, United States) using an unpaired Student's t-test or two-way ANOVA followed by Sidak's or Tukey's multiple comparison test. All data presented as mean ± SEM. Values outside two standard deviations from the mean were excluded.

### Ethics

Animal experimentation (microvessel isolation) was approved by the Animal Ethics Committee of the University of Queensland (approval numbers QBI/348/17/NHMRC and QBI/554/17/NHMRC). The animals were housed in specific pathogen-free cages and maintained on a 12 h light/dark cycle, with unlimited access to food and water.

## Results

### Establishment and characterization of eGFP-hCldn5-MDCK II cells

To manipulate BBB opening *in vitro*, we first evaluated a range of cell lines which are routinely used for these purposes. Of these, human iBECs are a suitable *in vitro* system because they form claudin-5-containing TJs, display a cobblestone-like morphology and have a high TEER (in the order of 4,000 Ω·cm^2^) 31, but the challenge is to ensure a constant supply of these cells. MDCK II cells, on the other hand, have been reported to display a much lower TEER of up to 200 Ω·cm^2^ 51-53. They express TJ proteins such as occludin and ZO-1, as well as several claudins including claudin-1, claudin-2 and claudin-4, but not claudin-5 54.

Using a lentiviral approach, we generated a stable eGFP-hCldn5-MDCK II cell line **(Figure 1B-C)**, establishing eGFP-expressing cells as a negative control. Both cell lines 53 displayed a cobblestone-like appearance, but whereas eGFP was localized to the cytoplasm in eGFP-MDCK II cells, the fusion protein was localized to the membrane in eGFP-hCldn5 MDCK II cells, as expected of a TJ protein **(Figure 1B-D)**. Importantly, claudin-5 did not localize to the plasma membrane when the cells were not in contact (i.e., when no TJs formed) **(Figure 1C)**. Claudin-5 could also be visualized with an anti-claudin-5 antibody, and the protein was found to be colocalized at cell-cell borders with both occludin and ZO-1, indicative of the protein being a TJ component **(Figure 1E-G)**.

**Figure 2:**
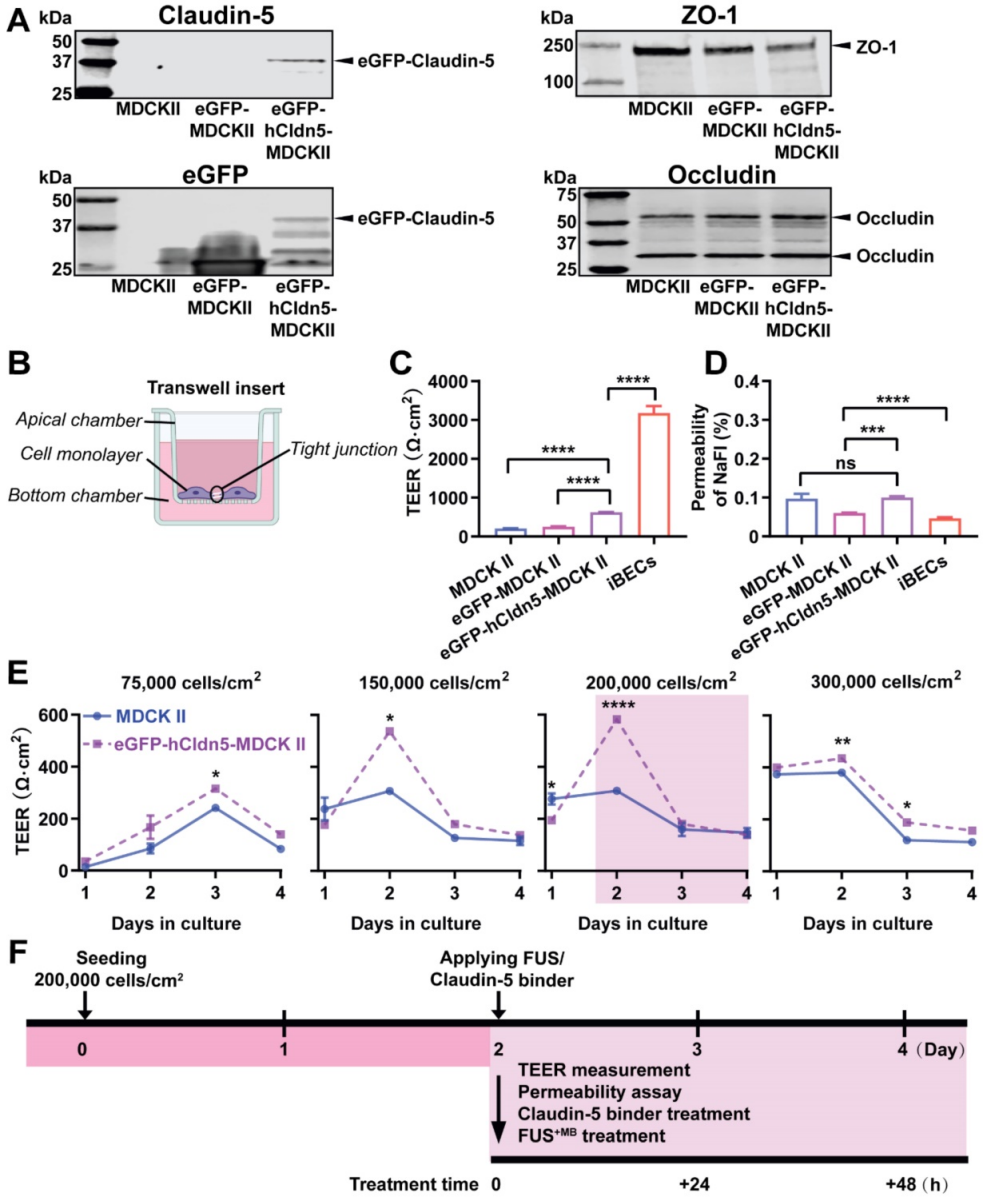
** eGFP-hCldn5-MDCK II cells exhibit a tight monolayer as determined by TEER and cargo leakage. (A)** Western blotting with either a claudin-5 or GFP antibody reveals expression of the eGFP-hClaudin5 fusion protein in eGFP-hCldn5-MDCK II but not eGFP-MDCK II cells. Expression of the tight junction proteins ZO-1 and occludin is also shown.** (B)** Scheme of Transwell insert to measure TEER and permeability.** (C)** eGFP-hCldn5-MDCK II cells display a four-fold higher TEER than MDCK II cells. iBEC cells are included for comparison. **(D)** All three MDCK II cell lines show a < 0.2% permeability for sodium fluorescein (NaFl), indicating a tight BBB. **(E)** TEER of eGFP-hCldn5-MDCK II and MDCK II cells shown as a function of cell density and days in culture. Asterisks refer to TEER differences between eGFP-hCldn5-MDCK II and MDCK II for a given time point. TEER values are shown as Ω·cm^2^ and results are expressed as mean ± SEM. N>10 per condition. (**F**) Schematic illustration of the experimental workflow. Statistical significance was determined as unpaired Student's t-test (*p<0.05, **p<0.01, ***p<0.001 and ****P<0.0001).

As a further validation, we performed a western blot analysis of eGFP-hCldn5-MDCK II, eGFP-MDCKII and untransfected cells, using antibodies for claudin-5, eGFP, occludin and ZO-1 **(Figure 2A, Figure S3)**. The cells expressed several LMW forms of occludin (major bands of 58 and 31 kDa) and ZO-1 (250 kDa). With eGFP having a molecular weight of 27 kDa and claudin-5 of 17 kDa, this revealed a claudin-5 fusion protein of an apparent molecular weight of approximately 44 kDa.

Next, we determined the TEER of eGFP-hCldn5-MDCK II cells compared to the parental MDCK II cell line and iBECs, culturing the cells on Transwell inserts. The TEER of iBECs was measured to be 3,450 Ω·cm^2^, consistent with what has been previously reported 31. Importantly, the mean TEER of eGFP-hCldn5-MDCK II cells was 622.9 ± 129.1 Ω·cm^2^, i.e., 3-fold higher than that of MDCK II cells (206.8 ± 49.9 Ω·cm^2^) **(Figure 2B-C)**. Of note, the TEER of eGFP-MDCK II control cells was slightly higher than for untransfected cells, possibly reflecting the fact that eGFP-MDCK II tended to overgrow, which was neither observed for the parental MDCK II cells nor for eGFP-hCldn5-MDCK II cells. The permeability characteristics of the eGFP-hCldn5-MDCK II and MDCK II cells were also determined by measuring the paracellular permeability of the cells grown on Transwell inserts to NaFl. This analysis revealed that less than 0.2% of NaFl (sodium fluorescein, MW 376 Da) passed to the lower chamber of the Transwell insert after 2 h, indicating a high level of barrier integrity **(Figure 2D)**. Of note, expression of hCldn5 did not further decrease permeability of NaFl possibly owing to the contribution of endogenously expressed claudin-2 which is more leaky 54. We also found that the TEER value was a function of cell density (testing four seeding densities ranging from 75,000 to 300,000 cells/cm^2^) and days in culture in that the highest TEER was obtained at 2 days in culture for cells seeded at 150,000 or 200,000 cells/cm^2^** (Figure 2E)**. We thereby established a model that allowed us to explore barrier function by measuring the TEER and permeabilities of the monolayer. For all subsequent experiments, cells were consistently plated at a density of 200,000 cells/cm^2^ and treated 2 days after plating **(Figure 2F)**.

### cCPEm and mC5C2 display a different time course of barrier opening in eGFP-hCldn5 MDCKII cells

**Figure 3:**
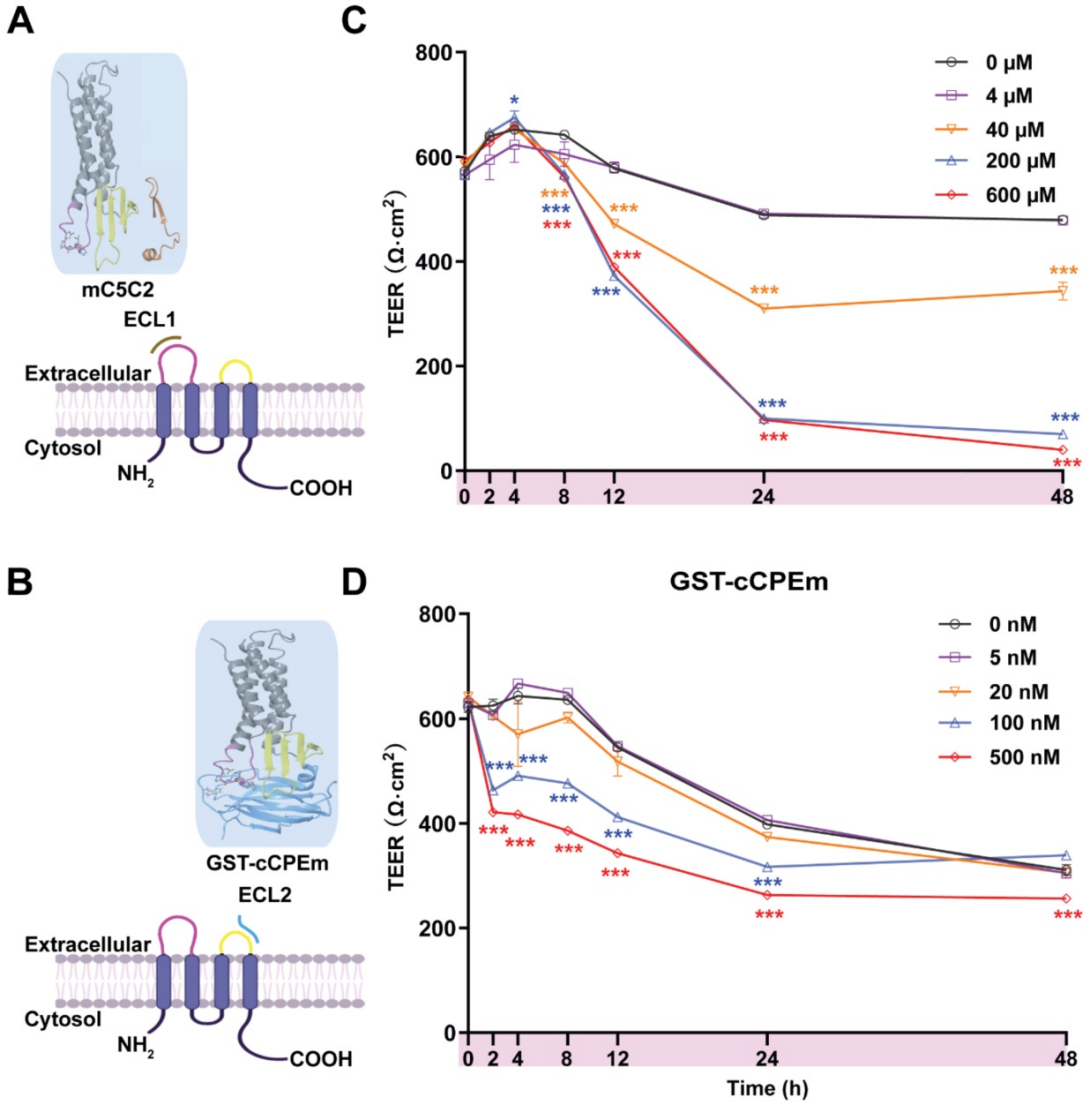
** Incubation with mC5C2 and GST-cCPEm reveals differences in the reduction of the absolute TEER in eGFP-hCldn5-MDCK II cells. (A-B)** Schematic diagram showing mC5C2 binding of the extracellular loop 1 (ECL1) and cCPEm binding ECL2 of claudin-5. The homology model of claudin-5 was created in Swiss-Model using human claudin-9 (PDB ID 6OV2) as template. The cCPEm structure was extracted from the same PDB entry (6OV2). mC5C2 was placed in proximity to the model of claudin-5 whereas cCPEm was docked to claudin-5 for schematic purposes only. The molecular structures were generated using Maestro (Schrödinger Release 2020-4, New York, 2020). **(C-D)** Incubation of eGFP-hCldn5-MDCK II with mC5C2 and GST-cCPEm causes concentration- and incubation-time-dependent reductions in TEER. N=6 of each condition. Two-way ANOVA with Sidak's multiple comparison test (*p<0.05, and ***p<0.001). Asterisks refer to the difference in TEER value at the measured time points, compared to the TEER value of untreated control.

The peptide mC5C2 binds to ECL1 with a reported nanomolar affinity** (Figure 3A)**, and the modified toxin GST-cCPEm has been reported to bind to ECL2 of claudin-5 with nanomolar affinity **(Figure 3B)** 35. We synthesized cCPEm and generated a fusion protein with cCPEm being carboxy-terminally fused to GST. As we did not remove the GST moiety, this allowed us to visualize binding to the TJs using an anti-GST antibody. By isolating murine cerebral microvessels **(Figure S4A-D)** we had a specimen in which we were able to observe BBB binding of GST-cCPEm, which colocalized with mCld-5 **(Figure S4E)**. mC5C2 was unlabelled so its binding could not be visualized. To determine how cCPEm and mC5C2 weaken the BBB, we tested the effects of different concentrations of the binders in time-course experiments in eGFP-hCldn5-MDCK II cells grown to confluency in 6.5-mm Transwell insets. A viability test proved that the two binders did not cause overt cytotoxicity **(Figure S5)**.

We tested mC5C2 at 0, 4, 40, 200 and 600 μM, and GST-cCPEm at 0, 5, 20, 100 and 500 nM concentrations, measuring both absolute TEER (in Ω·cm^2^) at 0, 2, 4, 8, 12, 24 and 48 h of incubation **(Figure 3C-D)** and relative TEER **(Figure S6)**. Compared with the peptide-free medium control, the TEER of eGFP-hCldn5-MDCK II cells displayed a time- and concentration-dependent decrease following treatment with mC5C2 and GST-cCPEm **(Figure 3C-D)**. We observed a weakening of the BBB as measured by TEER for the mC5C2 peptide after an 8 h incubation, and for GST-cCPEm after 4 h, with some indication of a drop at the higher concentrations already after 2 h. Together, there was a clear concentration-dependent effect for both treatments. Of note, the drop in TEER was more pronounced for the peptide with no indication of BBB closure even after 48 h, whereas for GST-cCPEm, the drop in TEER was less pronounced.

### Focused ultrasound treatment with acoustic pressures of 0.3 and 0.4 MPa achieves reliable barrier opening

**Figure 4:**
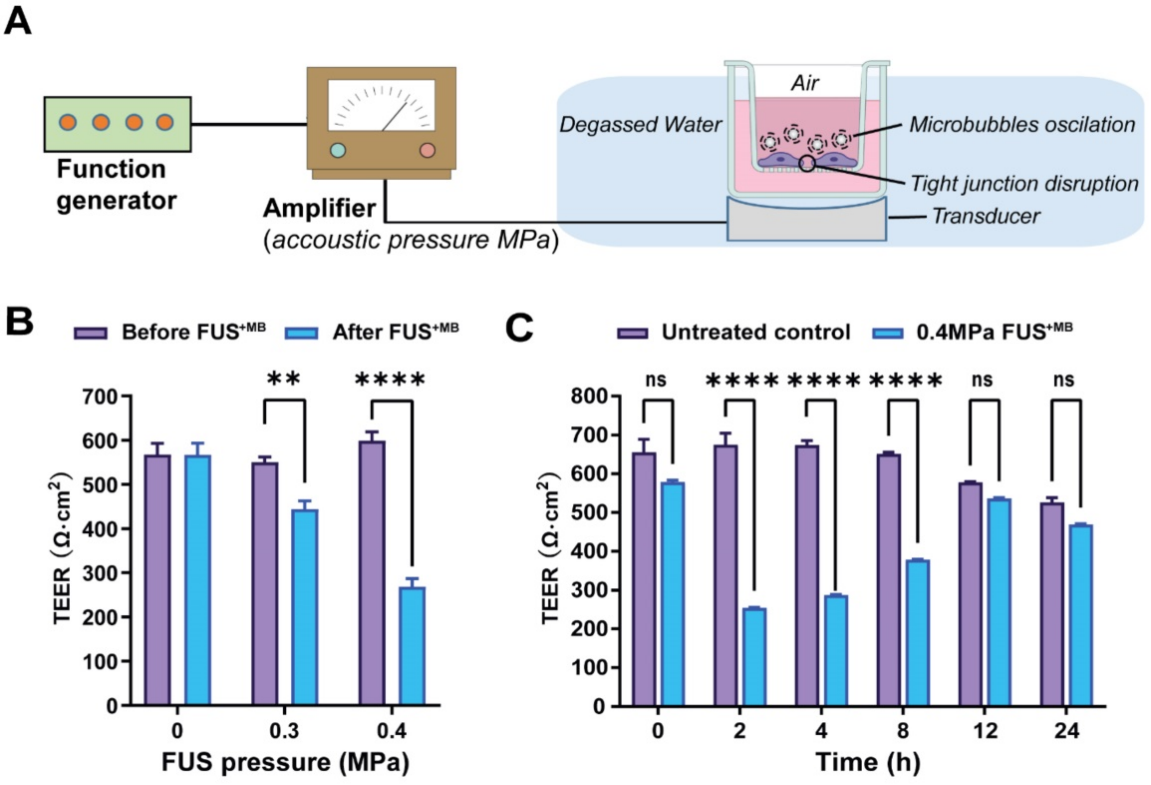
** Focused ultrasound with microbubbles (FUS^+MB^) leads to a rapid opening of the barrier followed by closure within 12 hours. (A)** Schematic diagram of how ultrasound is delivered to the cells. **(B)** TEER measurement as a function of acoustic pressure (in MPa) before and immediately after FUS^+MB^ treatment. **(C)** Absolute TEER measurement as a function of incubation time shown for the 0.4 MPa condition. N=3-6 for each condition. Two-way ANOVA with Sidak's multiple comparisons tests (**p<0.01 and ****P<0.0001).

We next explored FUS in the presence of microbubbles (FUS^+MB^) which is known to open the BBB in a time scale of seconds 55, 56 **(Figure 4A)**. The average diameter of MBs used in our study was 1.060 ± 0.64 µm, with a concentration of ~ 9.0 × 10^9^ MBs/ml **(Figure S7A)**. For iBEC cells, we had previously shown that cells detach from the monolayer at pressures of 0.3 MPa, and that at 0.15 MPa, barrier opening is achieved without destroying the monolayer and creating larger holes 31. MDCK II cells are more robust, which is why we tested a range of slightly higher pressures (up to 0.4 MPa). We found that at 0.3 and 0.4 MPa, the barrier was opened as demonstrated by a reduction in absolute TEER **(Figure 4B),** without causing damage to the cell layer as demonstrated with an MTT assay for all conditions **(Figure S5)**. To better compare the FUS^+MB^ data with those obtained for GST-cCPEm and mC5C2, we next used the same time points as above (0, 2, 4, 8, 12, and 24 h) to better understand barrier opening and closure. By determining the absolute **(Figure 4C)** and relative changes to TEER **(Figure S7B)** this revealed that the barrier closed within 12 h for the conditions tested.

### Preincubation with the BBB binder cCPEm enhances focused ultrasound-mediated barrier opening

**Figure 5:**
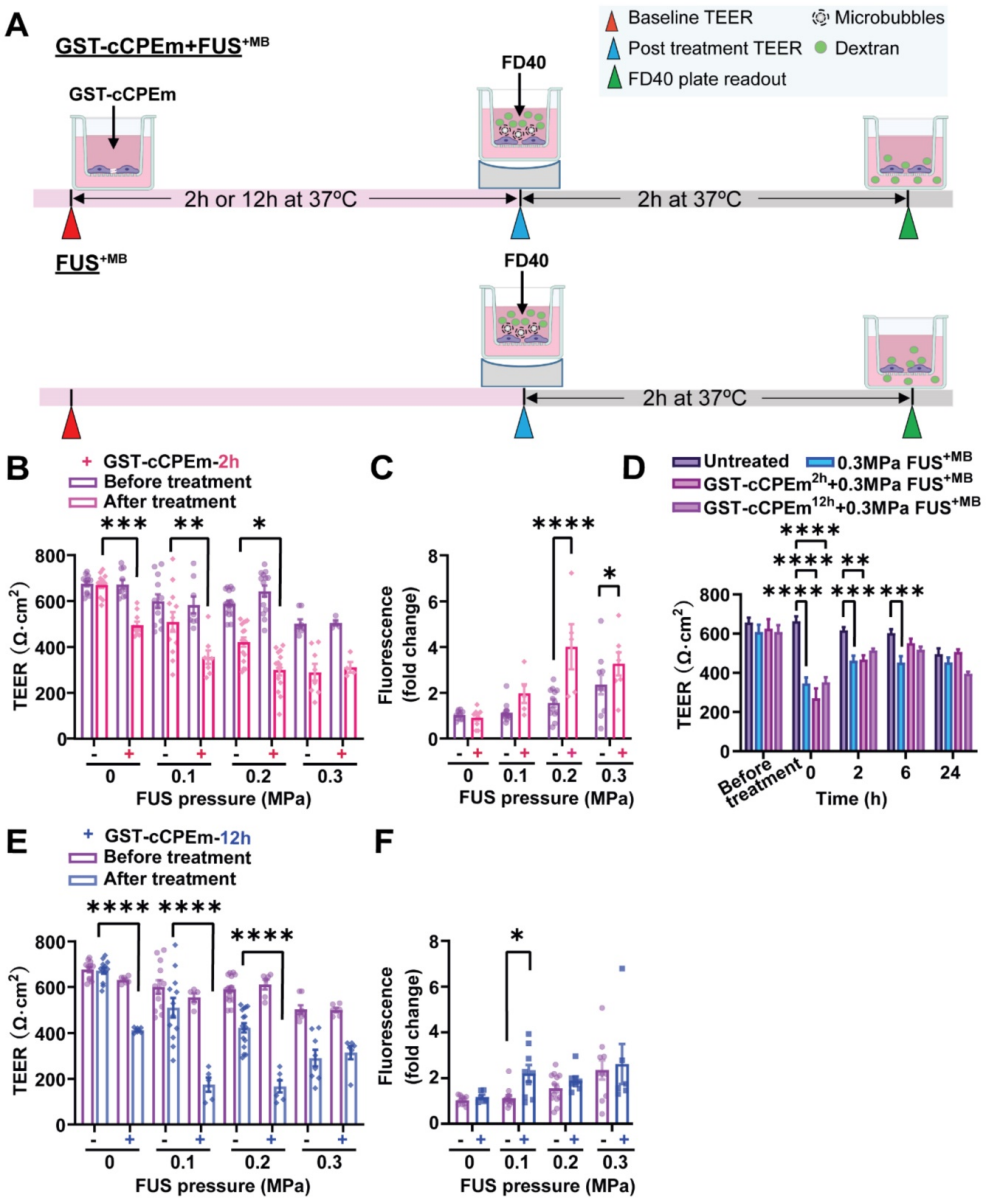
** Preincubation with GST-cCPEm lowers the acoustic pressure required for focused ultrasound-mediated barrier opening. (A)** Experimental scheme. **(B)** Absolute TEER measurement and **(C)** FD40 leakage of eGFP-hCldn5-MDCK II cells preincubated with 100 nM GST-cCPEm for 2 h, followed by FUS^+MB^ at the indicated pressures. **(D)** TEER measurement to assess barrier closure when GST-cCPEm was removed followed treatment. **(E)** Absolute TEER measurement and **(F)** assessment of FD40 leakage of eGFP-hCldn5-MDCK II cells preincubated with 100 nM GST-cCPEm for 12 h, followed by FUS^+MB^ at the indicated pressures. N≥5 from at least two independent experiments. Two-way ANOVA with Tukey's and Sidak's multiple comparisons tests (*p<0.05, **p<0.01, ***p<0.001 and ****P<0.0001).

We next examined whether pre-treatment with a BBB binder would lower the threshold required for FUS^+MB^ to achieve barrier opening and achieve a greater degree of opening at a pressure which results in weak opening in the absence of GST-cCPEm. Several parameters were explored to narrow down suitable conditions 31. Given that GST-cCPEm, at the concentrations used, achieved barrier opening faster than mC5C2 as measured by TEER, we pre-incubated the eGFP-hCldn5-MDCK II cells with 100 nM GST-cCPEm for 2 h, followed by FUS^+MB^ at 0.1, 0.2 and 0.3 MPa. GST-cCPEm and FUS^+MB^ only conditions were included as controls **(Figure 5A)**. TEER was determined at the start of the experiment (0 h, +/- GST-cCPEm) and after 2 h (+/- sonication). Leakage of 40 kDa FITC-labelled dextran FD40 was also determined after 2 h. We found that for all conditions, TEER was reduced compared to the untreated control.

At 0.3 MPa, GST-cCPEm had no impact on the extent of TEER reduction; however, by progressively dropping the acoustic pressure to 0.2 and then 0.1 MPa, the TEER reductions became more pronounced, with an additional 43.7% and 37.7% TEER reduction after preincubation with GST-cCPEm at the 2 h timepoint. A two-way ANOVA revealed a significant effect of FUS^+MB^ F(3,75)=45.29, p<0.0001 and a significant effect of GST-cCPEm on TEER F(1,75)=43.47, p<0.0001 **(Figure 5B)**. The changes in TEER were reflected by changes in permeability as determined for FD40 at 2 h post FUS^+MB^, which was increased by 158.7% and 73.6% when pre-incubated with GST-cCPEm compared with FUS^+MB^ alone at 0.2 MPa and 0.1 MPa, respectively. A two-way (FUS^+MB^ pressure) x (GST-cCPEm) ANOVA on FD40 leakage found a significant main effect of FUS^+MB^ (F_3,63_=17.49, p<0.0001). There was a significant main effect of GST-cCPEm on FD40 leakage (F_1,63_=23.72, p<0.0001) indicating GST-cCPEm increases the permeability of the monolayer to FD40 separately to the effect of FUS^+MB^. The interaction was significant (F_3,69_=5.74, p=0.0015) indicating that the effect of GST-cCPEm is different depending on the ultrasound pressure applied with leakage being enhanced by GST-cCPEm most successfully at 0.2 MPa FUS^+MB^ (p<0.0001) and also at 0.3 MPa FUS^+MB^ (p<0.03 Sidak multiple comparisons test) **(Figure 5C)**. Importantly, to explore whether the barrier integrity of treated monolayers could be restored, we removed the GST-cCPEm from the cell medium after the FUS treatment and monitored TEER for 24 h. No significant difference in absolute TEER values was observed between treated groups and the untreated control after 24 h **(Figure 5D).** The cell monolayer integrity was restored with TEER reaching around 85% of the baseline value after 24 h.

We then extended our study and prolonged the incubation time for the toxin to 12 h. Our results revealed that at a pressure of 0.1 and 0.2 MPa, when the eGFP-Cldn5-MDCK II cell monolayers were preincubated with GST-cCPEm for 12 h, the extent of TEER reduction for the combination treatment was a further 166.5% and 160.6%, compared with cells treated with 0.1 and 0.2 MPa FUS^+MB^, respectively. A two-way (FUS^+MB^ pressure) x (GST-cCPEm) ANOVA on the TEER recorded at 12h post FUS^+MB^ found there was a significant main effect of FUS^+MB^ such that there was a decrease in TEER with increasing pressure (F_3,63_ = 23.07, p<0.0001). There was a significant main effect of GST-cCPEm incubation revealing that preincubation with GST-ccCPEm lowers the TEER separately to the effect of FUS^+MB^ (F_1,63_ =73.61, p<0.0001). There was a significant FUS^+MB^ x GST-cCPEm interaction such that GST-cCPEm had a greater effect on TEER at low ultrasound pressures which was confirmed by Sidak multiple comparison's test which found that for each pressure the TEER was significantly lower when the cells were treated with combined FUS^+MB^ and GST-cCPEm indicating our hypothesis that GST-cCPEm would increase the amount of BBB opening at lower pressures of 0.1 MPa and above** (Figure 5E)**. The changes in TEER were reflected by changes in permeability in that the leakage of FD40 was increased by FUS^+MB^ (p=0.0017, two-way ANOVA) and GST-cCPEm (p=0.046, two-way ANOVA) **(Figure 5F).** Importantly, no damage to the cell layer was observed as demonstrated with an MTT assay for all combination treatment conditions after 48 h **(Figure S8C-D).**

**Figure 6:**
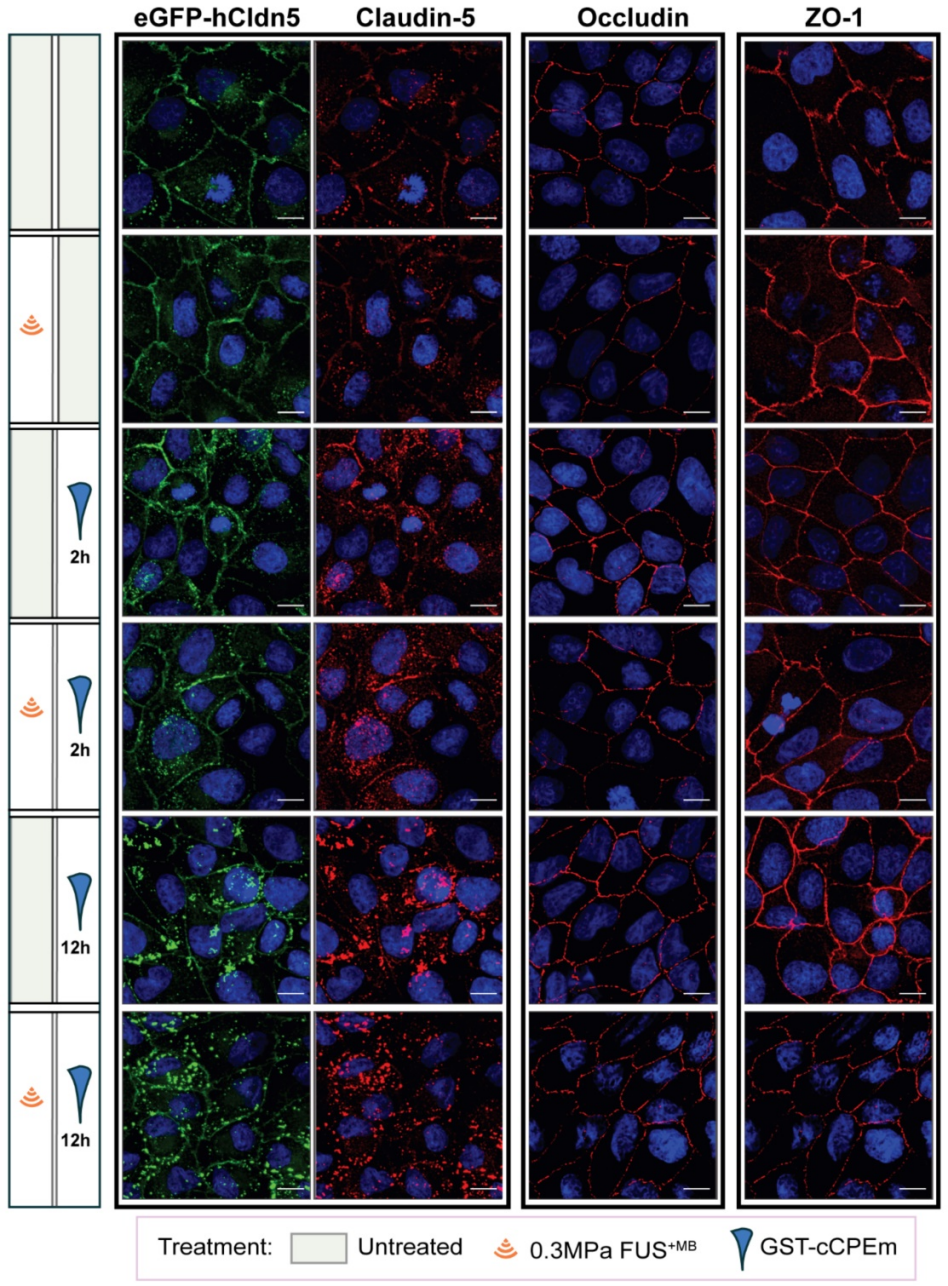
** Effects of GST-cCPEm and FUS^+MB^ alone and in combination on the junctional morphology of eGFP-hCldn5-MDCK II cells.** Immunostaining for claudin-5, occludin and ZO-1 for the indicated experimental conditions. The eGFP-hCldn5 and claudin-5 staining were performed in the same sample, whereas the occludin and ZO-1 staining were performed in separate samples. Staining of cell nuclei with DAPI. Scale bar =10 µm.

**Figure 7:**
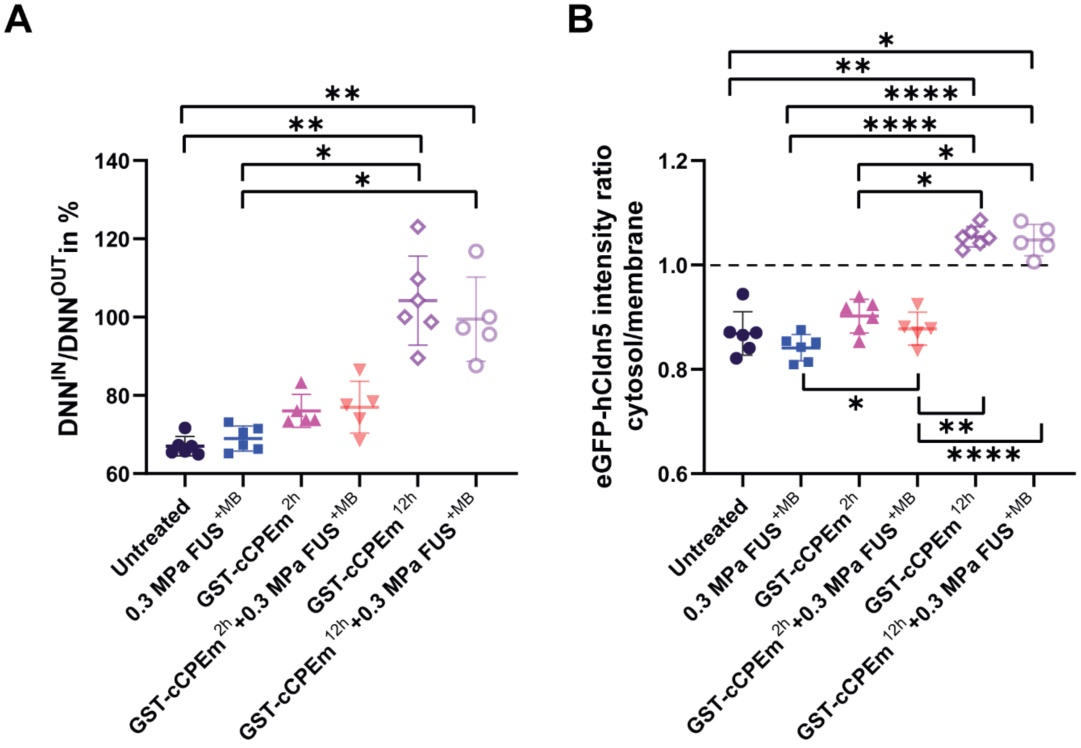
** Quantification of the redistribution of the eGFP-hCldn5 signal from the plasma membrane to the cytosol. (A)** The ratio of the nearest neighbour distance in the membrane (DNN^IN^) versus the nearest neighbour distance in the cytosol (DNN^OUT^) across conditions. The ratio was significantly higher for the 12 h GST-cCPEm and 12 h GST-cCPEm + FUS^+MB^ conditions, compared to the untreated control. One-way ANOVA with Tukey's multiple comparisons tests (*p<0.05 and **p<0.01). **(B)** The eGFP-hCldn5 intensity ratio in the cytosol compared to that of the membrane across conditions. One-way ANOVA with Tukey's multiple comparisons tests (*p<0.05, **p<0.01 and ****P<0.0001).

The question arises whether the changes in TEER and cargo leakage can be captured by microscopic analysis of key molecules such as claudin-5. We performed a confocal analysis and found that neither treatment caused drastic changes to occludin and ZO-1 **(Figure 6)** or the adherens junction protein, E-cadherin **(data not shown)**, indicating that cell-to-cell contacts were still present and that the monolayer did not disintegrate. We found that GST-cCPEm caused specific changes to claudin-5 reflecting its binding to this TJ protein. It appears that the immunolabelling at the cell borders was markedly decreased and that claudin-5 was relocated into the cytosol, particularly for the 12 h GST-cCPEm and 12 h GST-cCPEm + FUS^+MB^ conditions **(Figure 6)**. To quantify the relocation of the eGFP-hCldn5 signal from the membrane to the cytosol, we resorted to segmentation to measure the distances between objects and intensities of the underlying voxels **(Suppl. movie)**. The ZO-1 signal displayed a constant stability across all treatment conditions (**Figure S9A**). Thus, we relied on this signal to create a surface delineating the plasma membrane of the cells. On average, the volume of the membrane surface in the 202 × 202 × 13 µm field of view was 3,750 µm^3^, with no significant difference between the treatment conditions based on one-way ANOVA. We used the distance to the membrane surface as the main criterion to sort out the objects in the eGFP-hCldn5 channel. As shown in **Figure 7A,** the mean distance to the nearest eGFP-hCldn5 spot increased significantly in the membrane compared to the cytosol for the 12 h GST-cCPEm and 12 h GST-cCPEm + FUS^+MB^ combination conditions, reflecting the disappearance of eGFP-hCldn5 spots from the plasma membrane. This was supported by a reduction in the proportion of spots counted in the membrane (**Figure S9B**). **Figure 7B** shows the ratio of the eGFP-hCldn5 signal intensities in the cytosol versus the membrane. The eGFP-hCldn5 signal intensities were significantly lower in the cytosol compared to the membrane (ratio < 1) for all experimental groups except for the 12h GST-cCPEm and 12 h GST-cCPEm + FUS^+MB^ combination treatments, for which the intensity in the cytosol became brighter than in the membrane (ratio > 1). Taken together, the segmentation analysis shows that the eGFP-hCldn5 signal initially detected in the plasma membrane was relocated and clustered in the cytoplasm after 12 h GST-cCPEm and 12 h GST-cCPEm + FUS^+MB^ conditions. This analysis also reveals that a more refined analysis is required such as super resolution microscopy to pinpoint the subcellular changes in response to FUS^+MB^ and GST-cCPEm treatment 57.

Collectively, our data reveal that pre incubation with a BBB binder lowers the threshold for the acoustic pressure required to open TJs, suggesting that pre-treatment with a claudin-5 binder followed by FUS may achieve safe BBB opening *in vivo*.

## Discussion

Focused ultrasound with microbubbles (FUS^+MB^) is an emerging drug delivery technology for the treatment of brain diseases including AD and other neurological conditions by transiently opening the BBB 58. The aim of the current study was to determine whether barrier permeability can be increased by combining FUS^+MB^ with a claudin-5 binder such that in a clinical setting lower acoustic pressures could be used. It has been shown that even without delivering therapeutic agents, FUS^+MB^ can reduce signature protein aggregates in AD mouse models and improve memory and motor functions 48, 59, 60. Currently, this technology is being explored in several clinical trials in patients with AD, underscoring the potential utility of this emerging technology. Ultrasound is used in combination with intravenously injected MBs, which respond to the sound waves by going through repeated cycles of expansion and contraction 61. This causes multiple effects on brain endothelial cells including separation of TJs between adjacent cells, thereby achieving transient BBB opening which then allows uptake of blood-borne and other therapeutic agents by the brain in the focal zone of the ultrasound pressure field. However, given that the useful therapeutic pressure range is only within a rather narrow window, this poses an inherent risk of brain damage as can be identified by the presence of extravasated blood cells when high ultrasound pressures are applied 7-9. When the pressure is too low, there is little to no functional BBB opening and hence, cargo uptake is not effectively facilitated. With increasing acoustic pressure however, the BBB opens facilitating uptake of increasingly higher amounts of drugs by the brain, until a particular pressure threshold is reached, upon which the microbubbles undergo inertial cavitation which can cause damage to the vessels and the underlying brain tissue (which in addition also abrogates cargo uptake) 14. Agents which have been safely delivered into the brain parenchyma by FUS include dextrans of various sizes 4, 22, chemotherapeutic drugs such as doxorubicin or methotrexate 62, 63, antibodies in various sizes and formats 6, 64-66, and viral vectors used for gene therapy 67. Here, we established a simple cellular system and asked whether preincubation with compounds targeting TJ proteins such as claudin-5 would increase barrier permeability in response to a given FUS pressure to maximise the amount of drug that could potentially be delivered to the brain.

To explore this option, we first established an *in vitro* hCldn5-MDCK II cell culture system in which we validated two binders of claudin-5, mC5C2 and GST-cCPEm, both of which weaken the barrier over time. Compared with FUS, the two binders showed a different profile for the conditions tested by us. Whereas FUS caused rapid barrier opening followed by recovery after 12 h within the tested acoustic pressure range (0.1-0.4 MPa), GST-cCPEm achieved opening as measured by a change in TEER only after 2-4 h, and mC5C2 needed even longer (up to 8 h) to open the barrier within the examined concentration range. No closure was detected for mC5C2 within a time window of up to 48 h after treatment. Whether for GST-cCPEm there was a slight indication of recovery at around 48 h is difficult to conclude given a marked reduction in TEER beyond 24 h post treatment (72 h post seeding) because the culture medium was not changed, and cellular health deteriorates during these long incubation times (**Figure 3D**). Weakening of the barrier was assessed by measuring the TEER and transepithelial passage of fluorescent tracers. Although both are indicators of TJ integrity, they reflect different experimental parameters. The TEER reflects the ionic conductance of the paracellular pathway, whereas the flux of fluorescent tracers indicates paracellular water flow and is an indicator of the pore size of the TJs 28.

Our approach was guided by FDA guidelines which require a TEER threshold of at least 100 Ω·cm^2^ for any cell line used as a cell permeability tool 68. By generating an MDCK II cell line that expresses fluorescently labelled claudin-5, we demonstrated an optimally high TEER two days post-seeding, whereas Caco-2 cells, for example, require more than two weeks 69. We expressed human claudin-5 in MDCK II cells which are of canine origin as the canine claudin-5 sequence was not available. Another consideration was that, in a clinical setting, TJs express the human form of the protein. Murine claudin-5 has been used for the transfection of human brain endothelial (hCMEC/D3) cells, demonstrating integrity of the TJ strand network 70. However, human and murine claudin-5 are highly homologous (98.2%), and their two ECLs show a 100% homology between species 71. We would therefore assume no functional difference between human and murine claudin-5 transduced MDCK II cells with respect to barrier formation. An obvious limitation of our cellular system is that it lacks the other cell-types that form part of the NVU, such as astrocytes and pericytes. Also, the physics of an oscillating MB above a free monolayer differs substantially from that of a constrained capillary tube in the mammalian brain; however, future studies will determine the extent to which the findings obtained in a simple MDCK II cell system can be translated *in vivo*.

**Figure 8:**
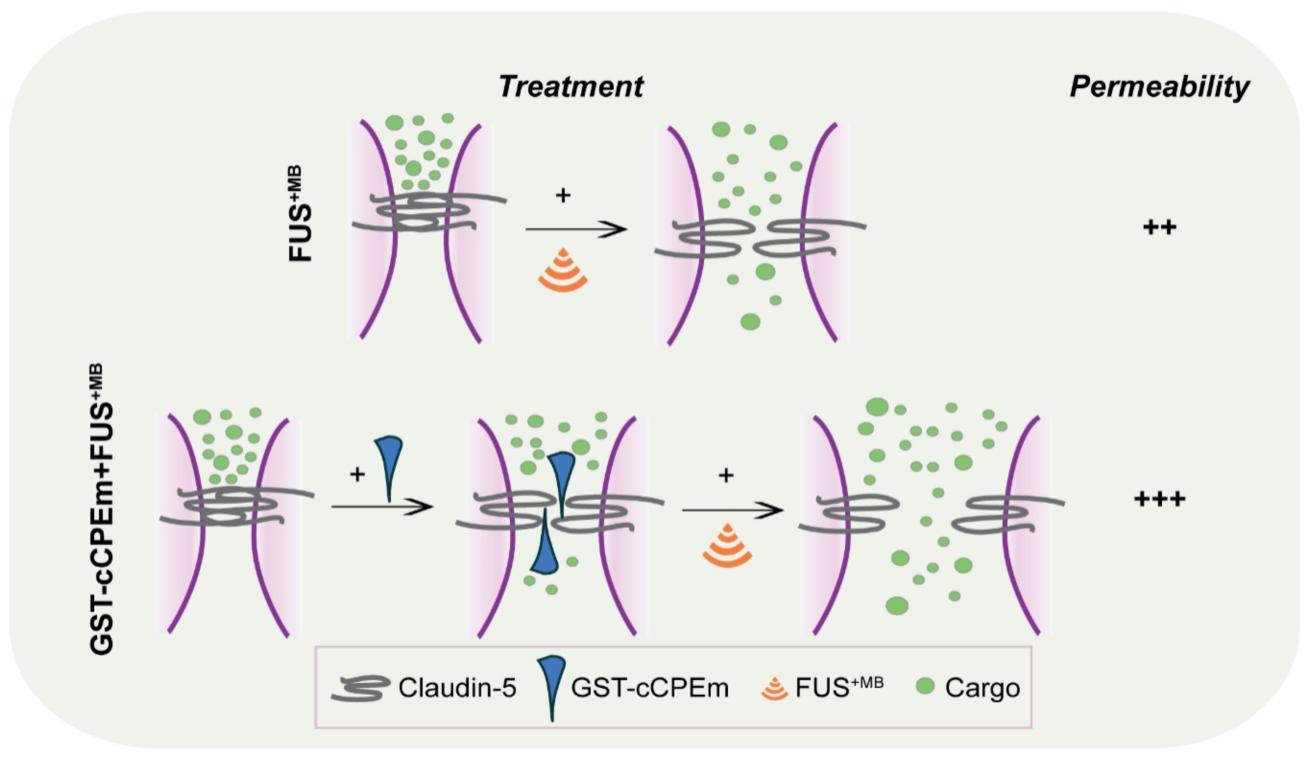
** Summary scheme of combinatorial treatment.** GST-cCPEm forms a wedge which destabilizes claudin-5 and thereby the TJs, making it easier for the ultrasound pressure wave to open the barrier, with potential implications for FUS-mediated drug delivery.

In an experimental or clinical *in vivo* setting, we foresee that the approach discussed here may be more generally applicable. Any peptide or small molecule compound that not only binds to but also weakens the interaction of TJ molecules such as claudin-5, or of adherens junction proteins, may be useful for enhancing the amount of drugs delivered by FUS as pre-weakening of the BBB by applying binders to the TJs increases the amount of drugs delivered to the brain by FUS. Alternative strategies could involve the combination of FUS with bradykinin (which increases the number of pinocytotic vesicles and hence facilitates transcytoplasmic transport), vasodilators such as papaverine (which reduces transcription and hence, the translation of proteins, including those that constitute TJs), or endothelial monocyte-activating polypeptide II (EMAP-II), which exerts a similar effect to papaverine 72-74. In our study we have not addressed transcytoplasmic transport, which is impacted by FUS in a cargo size-dependent manner 22. With regards to the effects on the transcription and translation of TJ proteins such as claudin-5, the effects observed in the aforementioned studies are the consequence of an activated signal transduction cascade which presumably has multiple effects. Our approach differs in that it targets TJ proteins directly. This is evidenced by our confocal analysis which revealed that claudin-5 is a direct target of cCPEm. without major changes to the monolayer. In our study, the binders to claudin-5 destabilize the barrier by forming a wedge **(Figure 8)**, lowering the requirements for ultrasound to open the barrier, which ultimately may allow for a more effective and safer uptake of therapeutic agents by the brain.

In an *in vivo* setting, the question arises how GST-cCPEm might be delivered in a clinical setting and whether the compound would persist in the circulation. A BBB binder such as GST-cCPEm could be administered intravenously or by intracarotid administration for enhanced local concentration in the brain vasculature prior to the FUS^+MB^ treatment 35, 39. In a supporting recent study, GST-cCPEm has been safely injected into larval zebrafish via the posterior cardinal vein, achieving increased leakage of a fluorescently labelled 10 kDa dextran up to 3 hours post-injection 75. Notably, fluorescence was detected in the vessel lumen with little leakage 4 hours post-injection. Suzuki and colleagues found that up to six repeated mucosal administrations of cCPE (that has a high affinity for claudin-3 and claudin-4) resulted in elevated cCPE-specific IgG in the serum of mice 76. Given that claudin-5 regulates the paracellular permeability for molecules below 800 Da *in vivo*, extravasation of GST-cCPEm (> 40 kDa) is unlikely to occur *in vivo* 77. We believe that widespread weakening of the BBB is unlikely to occur *in vivo* and that high levels of BBB opening and drug delivery will occur only where FUS is also applied, however this remains to be determined *in vivo*. Together, the safety, pharmacokinetic and antigenicity profiles of GST-cCPEm need to be evaluated in mammalian animal models prior to considering a clinical application. It seems therefore to be more relevant to determine dosing and treatment regimen for optimal opening e.g., in mice where the compound cannot be washed away.

In conclusion, we have generated a novel combinational strategy utilizing a claudin-5 specific binder, GST-cCPEm, together with FUS^+MB^ to weaken the BBB, and thereby enhance BBB permeability. The BBB permeability increase is reversible and safe *in vitro*. Through this combination strategy, we envisage that it helps paving the way for developing therapeutic ultrasound into a safe and effective treatment modality of diseases of the human brain.

## Supplementary Material

Supplementary figures and movie legend.

Supplementary movie.

## Abbreviations

BBB: blood brain barrier
FUS: focused ultrasound
MBs: microbubbles
TJs: tight junctions
TEER: transendothelial electrical resistance
CNS: central nervous system
NVU: neurovascular unit
BCECs: brain capillary endothelial cells
JAM: adhesion molecule
ESAMs: endothelial cell adhesion molecules
TM: transmembrane domains
ECL: extracellular loops
hiPSCs: human induced pluripotent stem cells
iBECs: human induced pluripotent stem cells derived endothelial cells
hCMEC/D.3: human cerebral microvascular endothelial cells
MDCK II: Madin-Darby Canine Kidney
FUS^+MB^: FUS, in conjunction with biologically inert gas-filled microbubbles
ECM: endothelial cell medium
GFAP: glial fibrillary acidic protein
PDGFRβ: platelet derived growth factor receptor β
TUJ1: class III β-tubulin
FD: Fluorescein isothiocyanate-dextran
NaFl: sodium fluorescein
DSPC: 1,2-distearoyl-sn-glycero-3-phosphocholine
DSPE-PEG2000: 1,2-distearoyl-sn-glycero-3-phosphoethanolamine-N- [amino (polyethylene glycol)-2000]
HPLC: high-performance liquid chromatography
MTT: 3-(4,5-dimethylthiazol-2-yl-2,5-diphenyltetrazoliumbromide
