# Supplementary material for: Claudin-5 binder enhances focused ultrasound-mediated opening in an in vitro blood-brain barrier model
Source: Theranostics. 2022 Jan 31;12(5):1952–70. doi: 10.7150/thno.65539 (PMC8899584; doi:10.7150/thno.65539)
Supplement: Supplementary file 1 — Supplementary figures and movie legend. [file thnov12p1952s1.pdf]

Supplementary Document

Figure S1

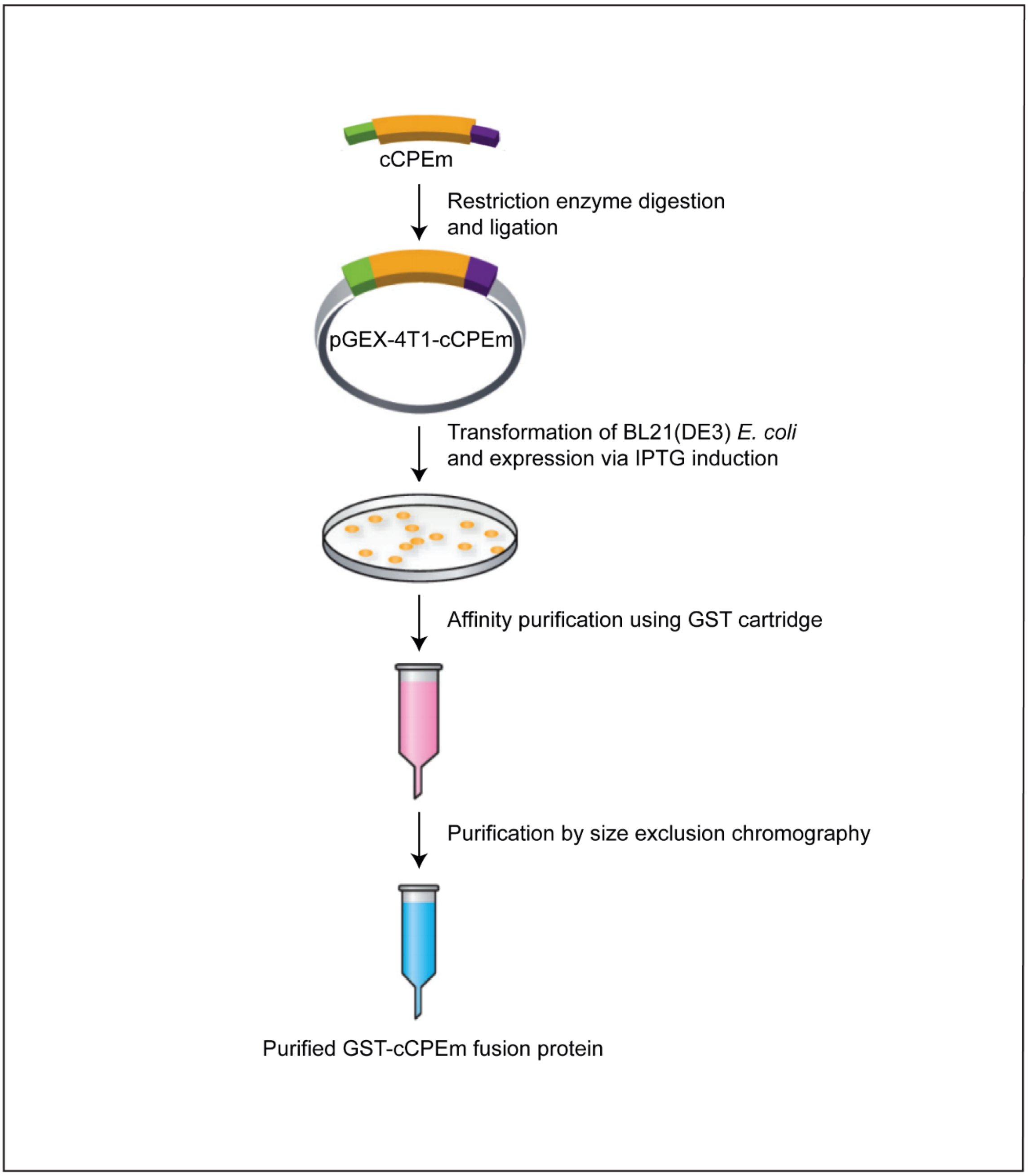

Figure S1: Schematic diagram of the generation of the GST-cCPEm fusion protein.
Created with BioRender.com

Figure S2

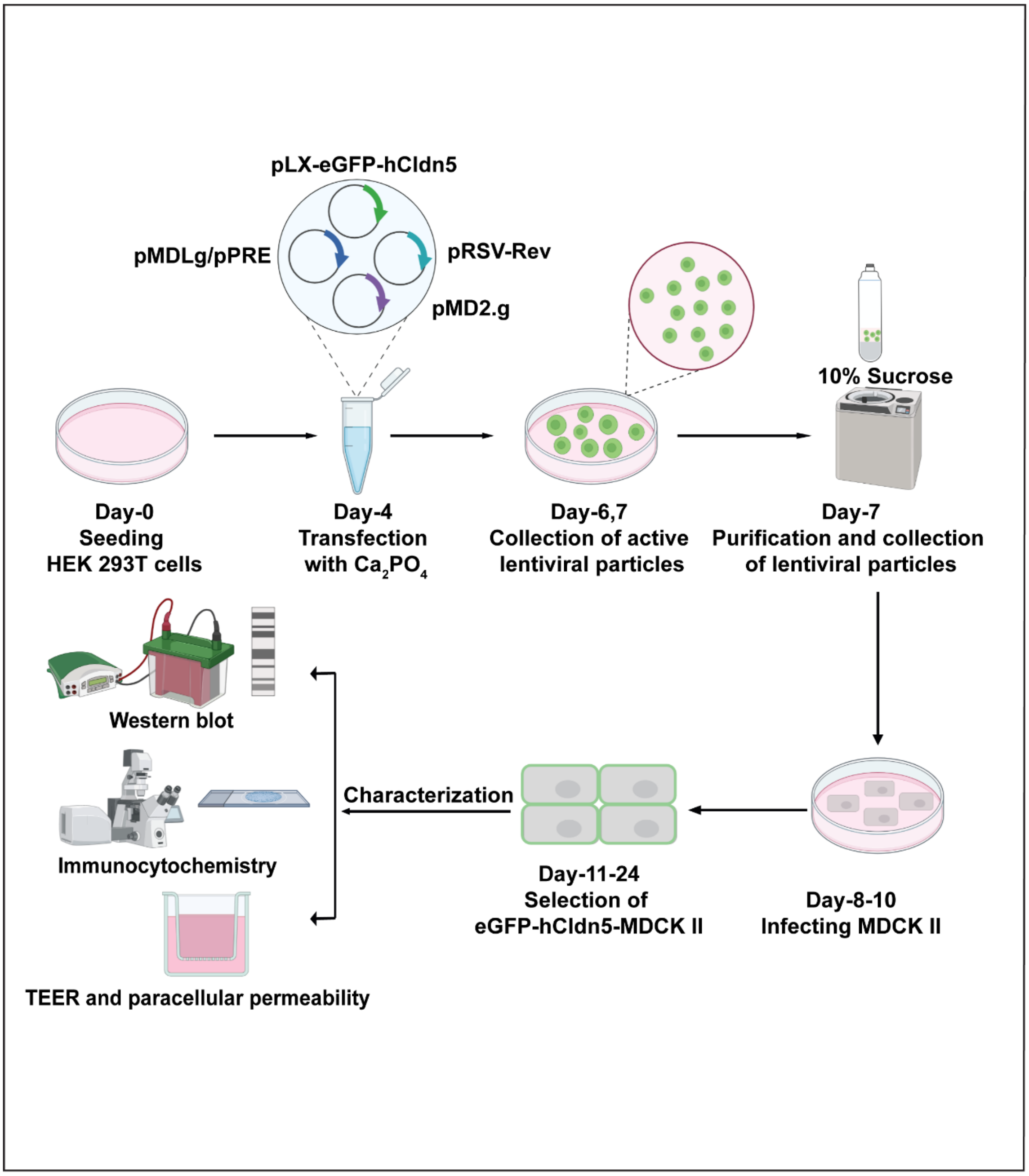

Figure S2: Schematic diagram of the generation of the stable cell line eGFP-hCldn5-MDCK II using lentiviral particles. Created with BioRender.com

Figure S3

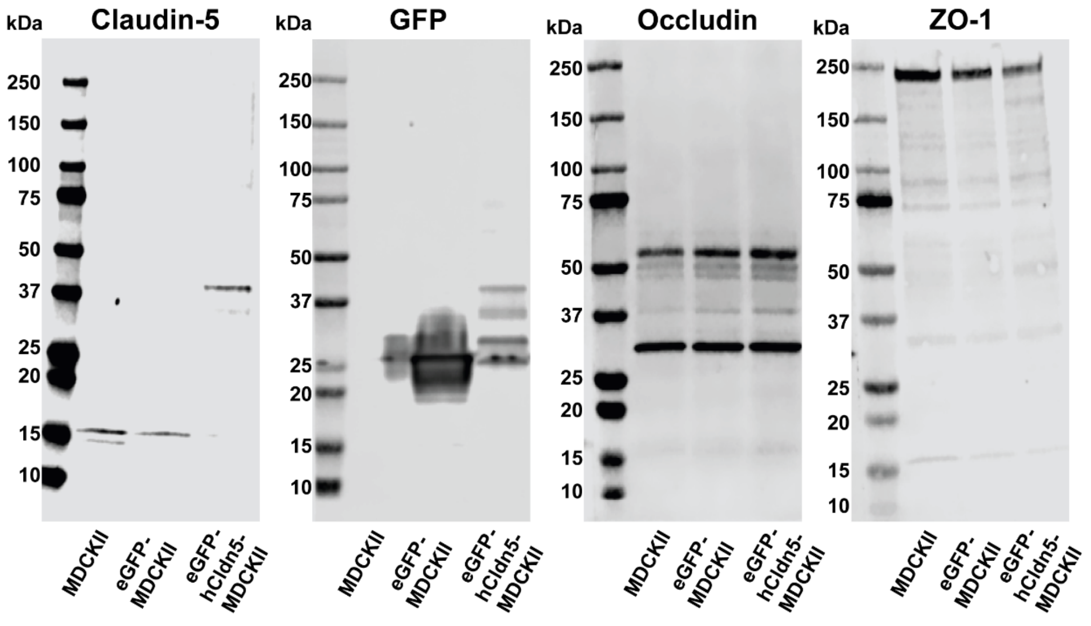

Figure S3: Western blot analysis of MDCK II, eGFP-MDCK II and eGFP-hCldn5-MDCK II cells for expression of the tight junction proteins claudin-5, occludin and ZO-1. Uncropped blots of what is shown in Figure 2A. All three cell lines express occludin and ZO-1. eGFP-hClaudin5 was only detected in eGFP-hCldn5-MDCK II cells. Note the unspecific band at 17 kDa in the claudin-5 blot for all conditions, and the flow-over of the sample in the GFP blot from the third into the second lane.

Figure S4

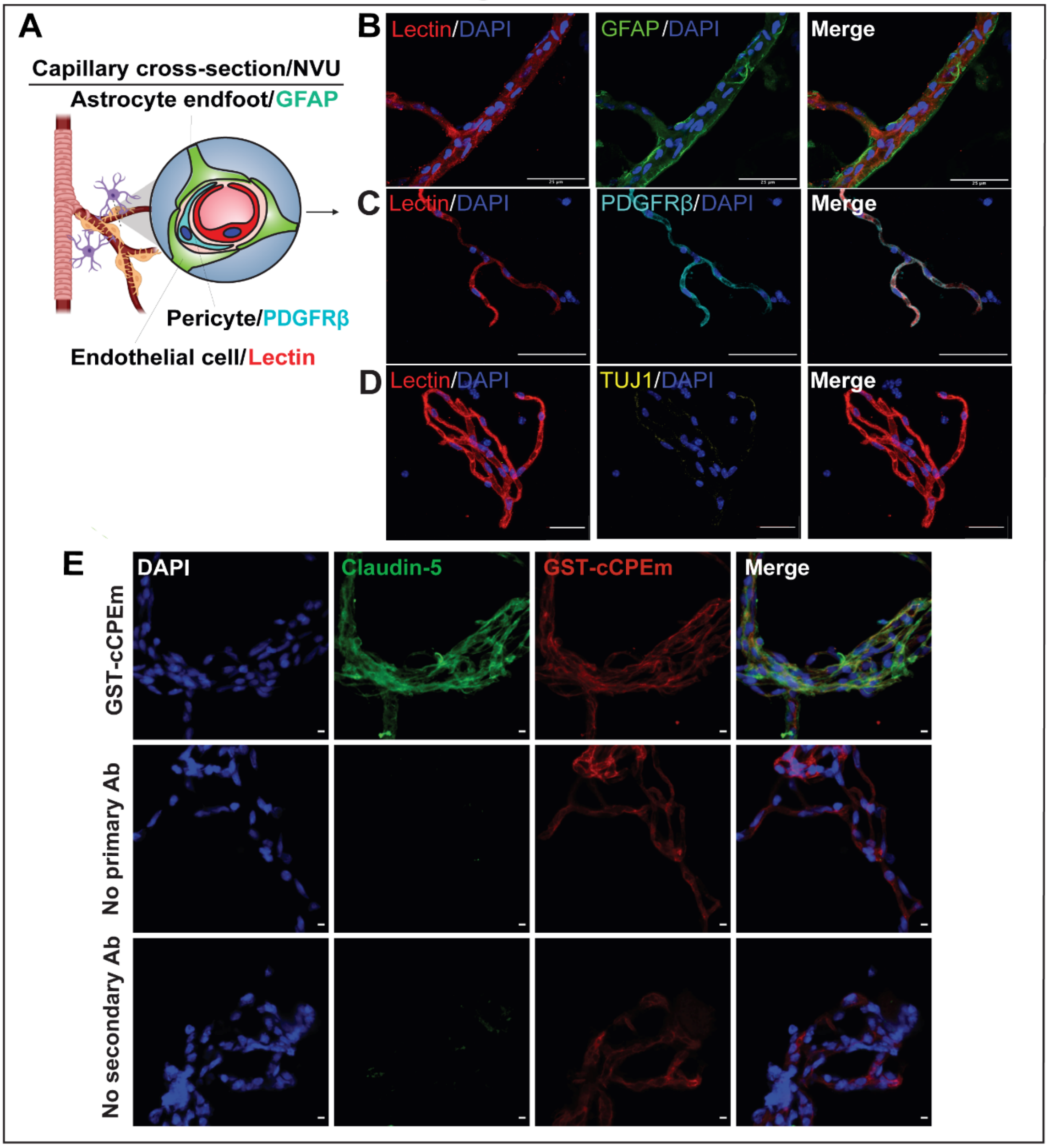

Figure S4: Characterization of *ex vivo* mouse brain microvessel explant preparations: cellular composition and purity. (A) Schematic of a representative mouse brain microvessel and cross-section showing the typical cellular components of the neurovascular unit (NVU). **(B-D)** Representative images of isolated mouse brain microvessels stained with antibodies for different cellular markers. **(B)** GFAP staining (green) indicating astrocytic end-feet that cover the outer surface of the cylindrical monolayer of cerebral microvascular endothelial cells, indicated by the lectin signal (red) at both the arteriole/venule level and the capillary level (white arrow). **(C)** Staining for PDGFRβ (cyan) indicates perivascular pericytes that also sheath the outer surface of capillary endothelial cells, indicated by the lectin signal (red). **(D)**. The absence of the neuronal marker TUJI (yellow) further confirmed the purity of the preparation. **(E)** GST-cCPEm bound to endogenous murine claudin-5 in isolated cerebral microvessels. Scale bar (B-D): 25 µm, (E): 5 µm.

Figure S5

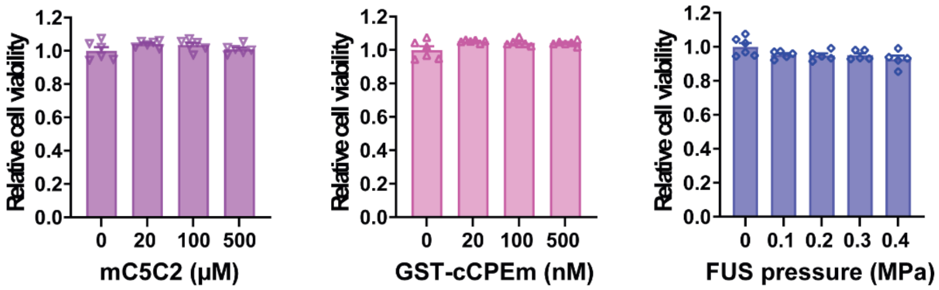

Figure S5: Cell viability analysis. (A) Cell viability of mC5C2- and **(B)** GST-cCPEm-treated eGFP-hCldn5-MDCK II cells. **(C)** Cell viability of eGFP-hCldn5-MDCK II cells after treatment with FUS^{+MB} for a pressure ranging from 0.1 to 0.4 MPa. Data are measured as fold change compared to the untreated samples and are presented as mean ± SEM. N≥5.

Figure S6

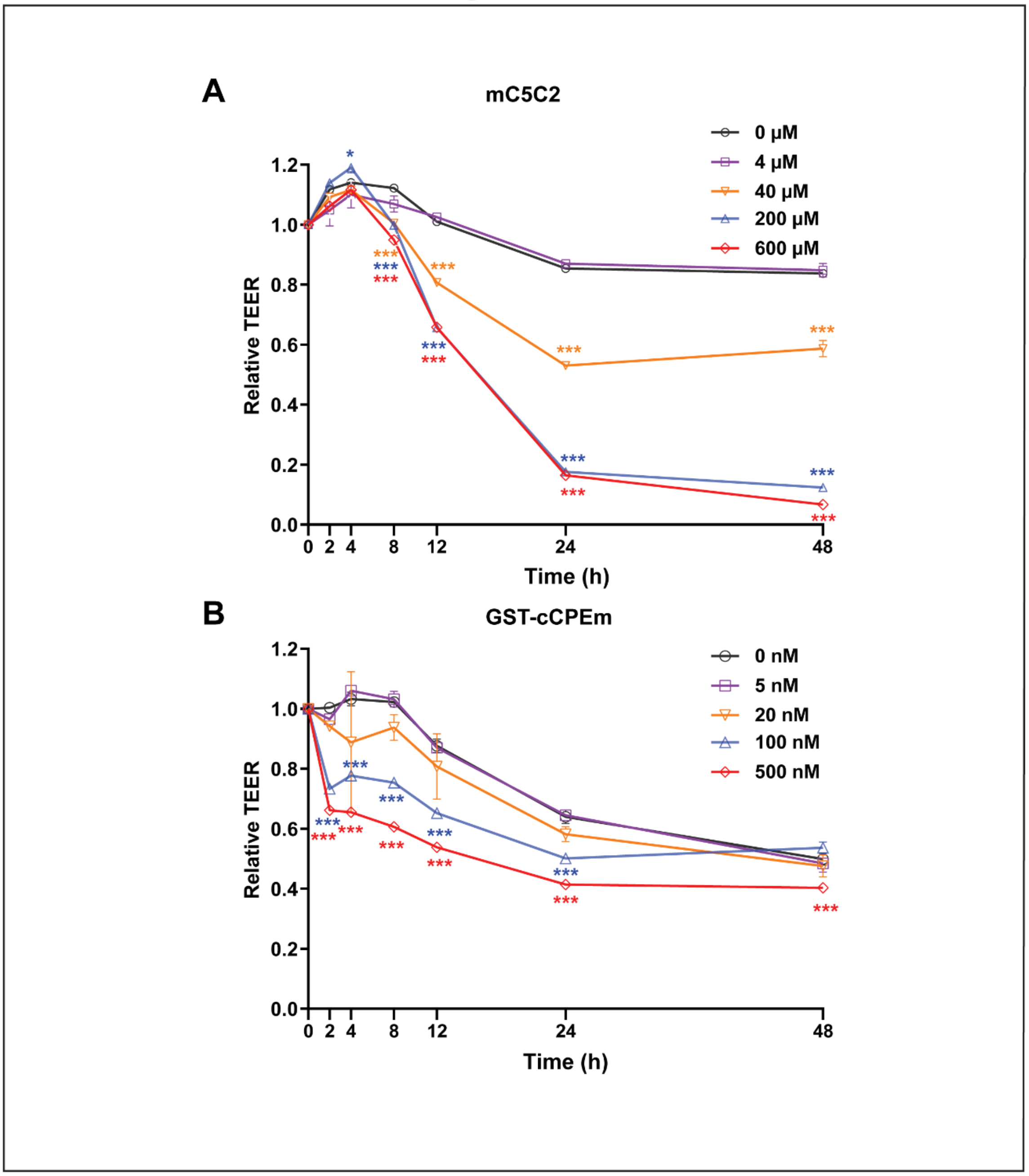

Figure S6: Relative TEER values: Incubation with mC5C2 and GST-cCPEm reveals differences in relative TEER reductions in eGFP-hCldn5-MDCK II cells. N=6 for each condition. Two-way ANOVA with Sidak's multiple comparison test (***p<0.001).

Figure S7

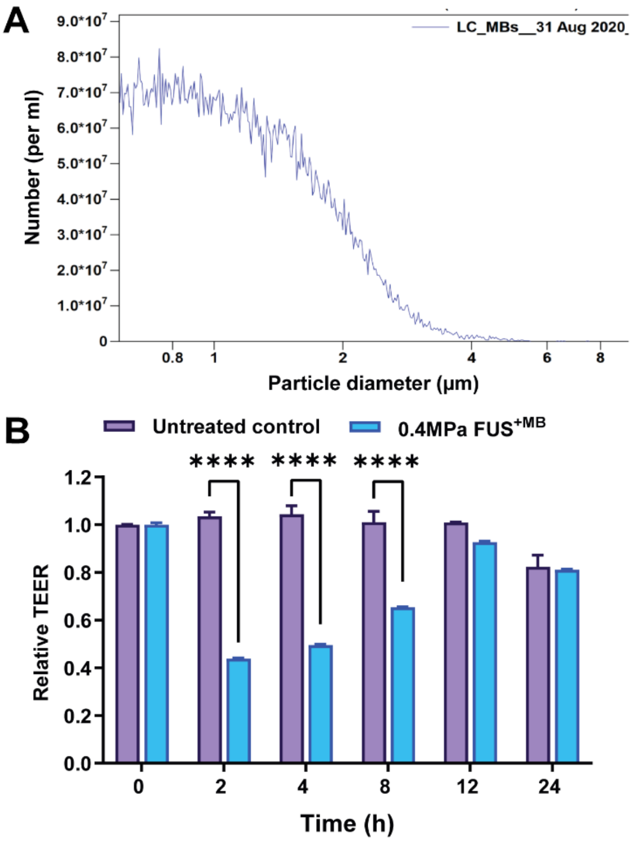

Figure S7: Microbubble quality control and absolute TEER values for FUS-treated eGFP-hCldn5-MDCK II cells. **(A)** Size and distribution profiles of in-house prepared biologically inert gas-filled microbubbles were measured with a Coulter counter. **(B)** Relative TEER values of FUS-treated cells.

Figure S8

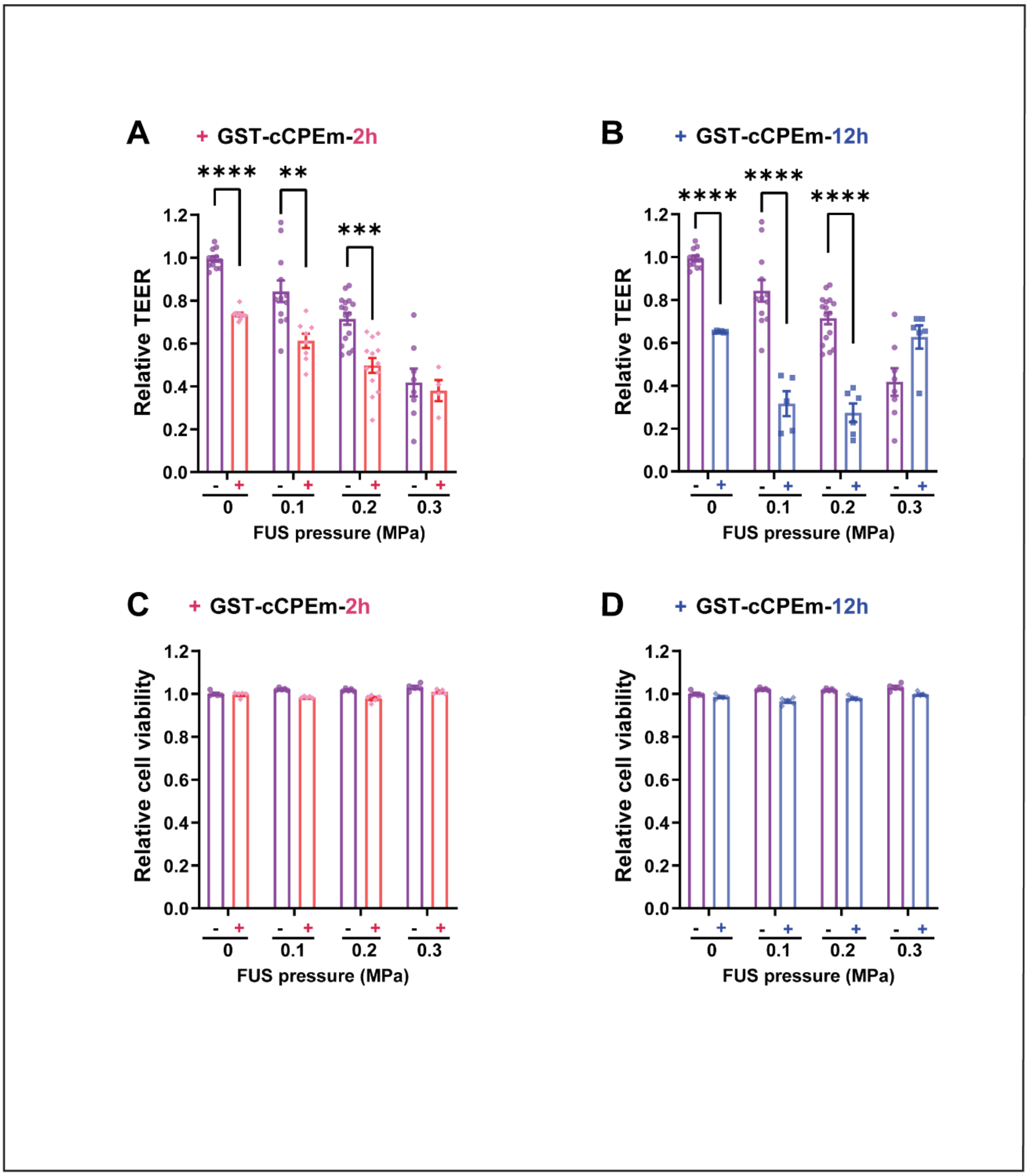

Figure S8: (A-B) Relative TEER values: Preincubation with GST-cCPEm lowers the acoustic pressure required for FUS-mediated barrier opening. N≥5 from at least two independent experiments. Two-way ANOVA with Tukey's and Sidak's multiple comparisons tests (**p<0.01, ***p<0.001 and ****P<0.0001). **(C-D) Cell viability evaluation of eGFP-hCldn5-MDCK II cells after combination treatment with GST-cCPEm and FUS^{+MB} for a pressure ranging from 0.1 to 0.3 MPa**. GST-cCPEm treatment was for 2 or 12 h, and cell viability was determined at 48 h. Data are measured as fold change compared to the untreated samples and presented as mean ± SEM. N=5**.**

Figure S9

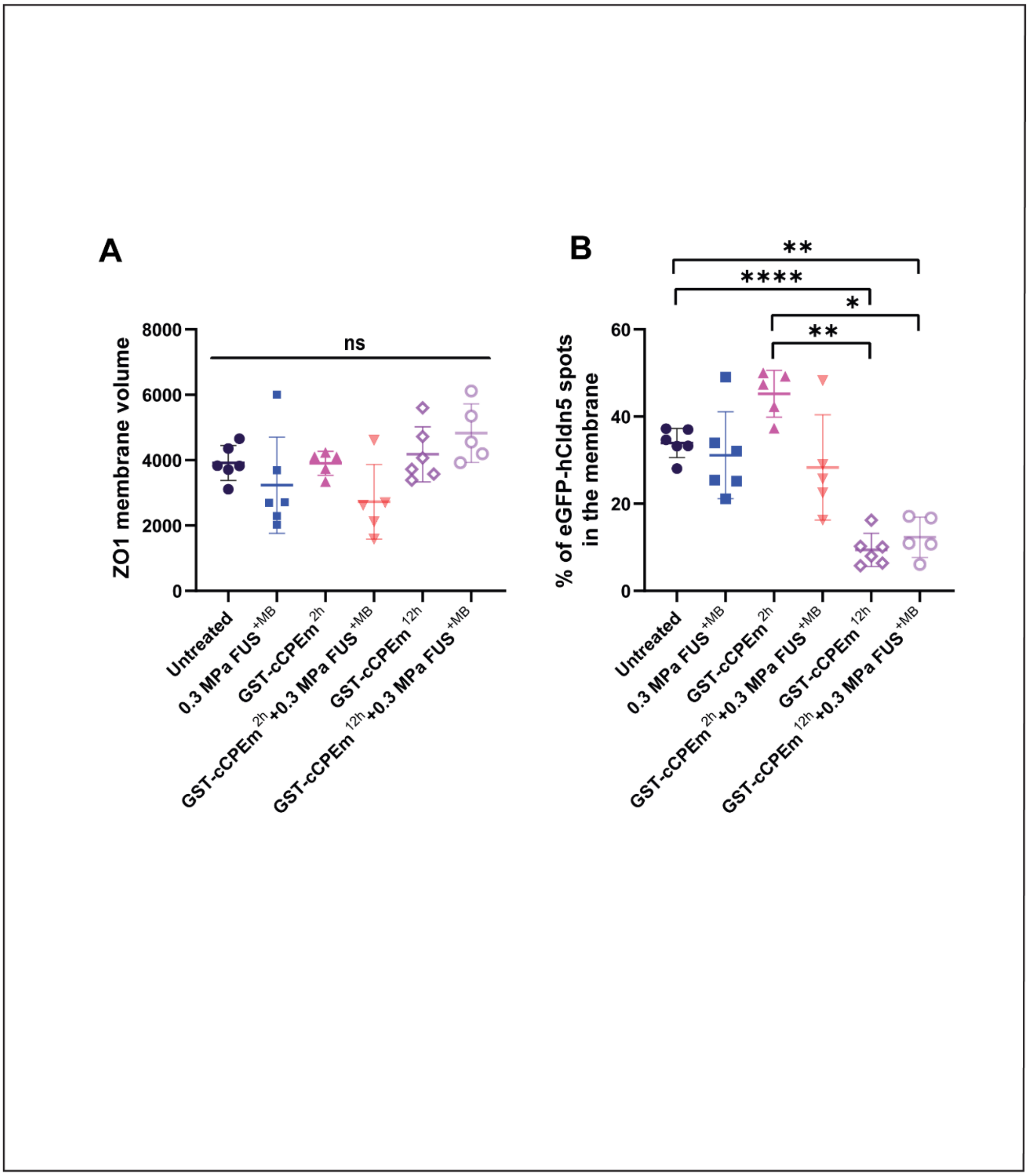

Figure S9: (A) ZO1 membrane volume for different conditions. Volumes were determined from the entire image stack (field of view 202 × 202 × 13 µm). No significant difference was found across treatments. **(B) Proportion of eGFP-hCldn5 spots detected in the membrane across treatments.** The proportion of eGFP-hCldn5 spots in the membrane decreased significantly after 12 h GST-cCPEm treatment and after the 12 h GST-cCPEm + FUS^{+MB} combination treatment**.** One-way ANOVA with Tukey's multiple comparisons tests (*p<0.05, **p<0.01, and ****P<0.0001).

Supplementary movie file

Suppl movie: Animation of the segmentation in Imaris. Two samples are juxtaposed for comparison, one untreated control (left) and one sample 12 hours after treatment with GST-cCPEm+0.3 MPa FUS^{+MB} (right). The processing steps are displayed contextually in the video. The original voxel data is displayed first, for all three channels acquired, i.e., DAPI (Ex=405 nm in blue), eGFP-hCldn5 (Ex=488 nm in green) and ZO1 (Ex=640 nm in red). The voxel data is then progressively replaced by the corresponding segmented models, starting with the membrane based on ZO1 in transparent red, the spots based on eGFP-hCldn5 in green, and the surfaces based on eGFP-hCldn5 in transparent white. The view then zooms in on a few cells to display more precisely the distribution of the spots inside the cells and then displays spots within 250 nm of the membrane surface in bright yellow, and the other in dull green. Notice the dramatic discrepancy in the number of spots in the membrane between both conditions. The same process is shown for surfaces based on eGFP-hCldn5, displaying the surfaces used for intensity measurement close to the membrane in solid white, the other in transparent white. The animation eventually reverts to displaying the voxel data. The process of segmentation displayed here is the source of all statistical analysis in Figure 7 across all samples.
